# Bidirectional association of sleep disorders with chronic kidney disease: a systematic review and meta-analysis

**DOI:** 10.1093/ckj/sfae279

**Published:** 2024-10-18

**Authors:** Jin Hean Koh, Claire Yi Jia Lim, Kvan Jie Ming Yam, Brian Sheng Yep Yeo, Adele Chin Wei Ng, Shaun Ray Han Loh, Pon Poh Hsu, Joshua Gooley, Chieh Suai Tan, Song Tar Toh

**Affiliations:** Yong Loo Lin School of Medicine, National University of Singapore, Singapore, Singapore; Faculty of Medical and Health Sciences, University of Auckland, Auckland, New Zealand; Lee Kong Chian School of Medicine, Nanyang Technological University, Singapore, Singapore; Yong Loo Lin School of Medicine, National University of Singapore, Singapore, Singapore; Department of Otorhinolaryngology-Head & Neck Surgery, Singapore General Hospital, Singapore, Singapore; Department of Otorhinolaryngology-Head & Neck Surgery, Singapore General Hospital, Singapore, Singapore; Signature Research Programme in Neuroscience & Behavioural Disorders, Duke-NUS Medical School, Singapore, Singapore; Yong Loo Lin School of Medicine, National University of Singapore, Singapore, Singapore; Signature Research Programme in Neuroscience & Behavioural Disorders, Duke-NUS Medical School, Singapore, Singapore; Department of Otolaryngology, Sengkang General Hospital, Singapore, Singapore; Signature Research Programme in Neuroscience & Behavioural Disorders, Duke-NUS Medical School, Singapore, Singapore; Yong Loo Lin School of Medicine, National University of Singapore, Singapore, Singapore; Signature Research Programme in Neuroscience & Behavioural Disorders, Duke-NUS Medical School, Singapore, Singapore; Department of Renal Medicine, Singapore General Hospital, Singapore, Singapore; Yong Loo Lin School of Medicine, National University of Singapore, Singapore, Singapore; Department of Otorhinolaryngology-Head & Neck Surgery, Singapore General Hospital, Singapore, Singapore; Signature Research Programme in Neuroscience & Behavioural Disorders, Duke-NUS Medical School, Singapore, Singapore

**Keywords:** renal failure, renal insufficiency, sleep apnea, sleep quality

## Abstract

**Background:**

Published studies have suggested a link between chronic kidney disease (CKD) and sleep disorders, although the exact nature of this association has not been uniformly described. Clarifying this relationship may facilitate evidence-based interventions that address the interplay between these disease entities. Such interventions could prevent obstructive sleep apnea (OSA) from worsening CKD and improve the quality of life for CKD patients by reducing the risk of developing OSA. Therefore, the objective of this meta-analysis is to assess the bidirectional association between sleep disorders and CKD.

**Methods:**

Following a PROSPERO-registered protocol, three blinded reviewers conducted a systematic review of the Medline/PubMed, Embase, Cochrane Library and Cumulative Index of Nursing and Allied Health (CINAHL) databases for observational studies pertaining to the relationship between sleep disorders and CKD. A meta-analysis was conducted in risk ratios (RRs).

**Results:**

From 63 studies (26 777 524 patients), OSA [RR 1.68; 95% confidence interval (CI) 1.45 to 1.93], albuminuria (RR 1.54; 95% CI 1.18 to 1.99), restless leg syndrome (RLS) (RR 1.88; 95% CI 1.48 to 2.38) and insomnia (RR 1.24; 95% CI 1.01 to 1.54) were significantly associated with CKD. There was a significant association between OSA (RR 1.77; 95% CI 1.56 to 2.01) with incident CKD. There was a significant association of OSA (RR 1.74; 95% CI 1.55 to 1.96), RLS (RR 1.73; 95% CI 1.32 to 2.25) and insomnia (RR 1.14; 95% CI 1.03 to 1.27) in patients with CKD compared with healthy controls. CKD was also significantly associated with incident OSA (RR 1.60; 95% CI 1.35 to 1.89).

**Conclusion:**

The bidirectional associations of obstructive sleep apnea with CKD remained consistent across different stages of CKD, modes of diagnosis of sleep disorder and geographical region. A bidirectional association was observed between CKD and obstructive sleep apnea, RLS and insomnia. The treatment of sleep disorders may reduce the risk of CKD, and vice versa.

KEY LEARNING POINTS **What was known:**

- Sleep disorders and chronic kidney disease (CKD) often co-exist. It is still unclear whether there is a bidirectional association between these two entities.

**This study adds:**

- This study highlights the relationships between sleep disorders and CKD.
- In addition to obstructive sleep apnea, this study also clarifies the bidirectional association between CKD and other sleep disorders, such as restless legs syndrome and insomnia.

**Potential impact:**

- Physicians treating patients with sleep disorders should be aware of this association with CKD and vice versa, and adopt measures targeted at addressing the co-existence of these disorders.

## INTRODUCTION

Sleep disorders are a growing global epidemic, and have been closely associated with chronic diseases such as type 2 diabetes mellitus, chronic kidney disease (CKD), cognitive dysfunction and neuropsychiatric disorders [1–4]. Patients with CKD are vulnerable to developing sleep disorders, which may lead to chronic fatigue and severely reduced quality of life. The reported prevalence of sleep disorders in patients with CKD ranges from 50% to 75% [5]. After accounting for direct and indirect costs, the annual costs related to sleep disorders is estimated to be approximately US$90 billion, representing a large financial burden to patients and policymakers [6].

Evidence for sleep disorders such as obstructive sleep apnea (OSA) and CKD have been reported since 1985 [7]. Poor sleep and insomnia may accelerate the progression of CKD and increase mortality among patients on maintenance dialysis [8]. On the other hand, patients requiring kidney replacement therapy may encounter relatively more frequent sleep disorders secondary to derangements of uremia metabolism [9]. In terms of OSA, the underlying mechanisms may point to OSA accelerating the decline in kidney function and the progression of a pre-existing CKD. OSA and CKD may also be linked in a bidirectional relationship; OSA may increase the risk of renal damage whereas CKD can in turn confer a higher risk of OSA [10]. Mechanisms by which CKD could predispose to OSA include uremia-induced neuropathy or myopathy, altered chemosensitivity and hypovolemia [10].

It is increasingly being recognized that sleep disorders and CKD are interrelated comorbidities. Besides a negative impact on sleep and quality of life, these disorders are associated with increased morbidity and mortality. A systematic review by Umbro *et al*. reported a significant association between OSA and CKD, particularly in more severe stages [11]. However, a meta-analysis was not performed, which precluded the quantification of the association between OSA and CKD. The aforementioned study was also restricted to only patients with OSA [11]. Other studies pertaining to other sleep disorders have reported only on the pooled prevalence of sleep disorders in patients with CKD, without being able to quantify the pooled risk of sleep disorders in patients with CKD and vice versa [12]. Therefore, the purpose of this systematic review and meta-analysis is to obtain up-to-date estimates of the risk of sleep disorders in patients with CKD and vice versa. The hypothesis of this study is that there exists a bidirectional relationship between sleep disorders and CKD.

## MATERIALS AND METHODS

### Data sources and searches

The protocol for this review was registered with PROSPERO (CRD42023423427). With reference to the Preferred Reporting Items for Systematic Review and Meta-Analyses (PRISMA) guidelines, a search was conducted on Medline/PubMed, Embase, Cochrane Register for Controlled Trials (CENTRAL) and Cumulative Index of Nursing and Allied Health (CINAHL) databases for studies published from inception to 29 September 2023 [13]. The search strategy used a combination of free text words and medical subject heading (MeSH) terms (Supplement 1). The reference lists of systematic reviews and included articles and the gray literature were also screened manually to identify additional studies for a comprehensive search. Contact with authors of included studies was made where feasible to collect supplementary data.

### Study selection

Two blinded reviewers (C.Y.J.L. and K.J.M.Y.) independently screened titles and abstracts to check the eligibility for inclusion using the online platform Rayyan, with disputes being resolved through consensus from a third independent author (J.H.K.). Reviewers then assessed the full texts of shortlisted studies against the following pre-defined inclusion and exclusion criteria.

The inclusion criteria were (i) observational studies that investigated the association between sleep disorders and CKD or albuminuria in participants aged 18 years or older, with comparison against a group of healthy controls, (ii) full-text studies, (iii) published in a peer-reviewed journal and (iv) published in the English language. In accordance with the published definitions of the National Kidney Foundation Kidney Disease Outcomes Quality Initiative (KDOQI), participants with CKD were defined as individuals with (i) an estimated glomerular filtration rate (eGFR) below 60 mL/min/1.73 m^2^ for 3 months or more, irrespective of cause, or (ii) having structural or functional abnormalities of the kidney for 3 months or more, with or without decreased GFR [14]. Albuminuria was defined as an albumin excretion rate ≥30 mg/24 h or an albumin:creatinine ratio of ≥30 mg/g. Studies investigating participants with renal replacement therapy (RRT) were included. The exclusion criteria were (i) studies including participants <18 years old, (ii) animal studies, (iii) case reports, (iv) *in vitro* studies and (v) reviews.

### Data collection

Data from included articles were collected by two blinded, independent reviewers (C.Y.J.L. and K.J.M.Y.) in duplicate onto a structured proforma specifically designed for the study and piloted beforehand on a sample of selected studies. Disagreement was resolved by discussion and consensus with a third reviewer (J.H.K.). Relevant study characteristics were collected on the data extraction spreadsheet, including but not limited to: geographical region, sample size, baseline characteristics of participants such as mean age, gender, ethnicity and mean body mass index, comorbidities such as diabetes and hypertension, definition of CKD, how CKD was assessed and stage of CKD. Relevant outcome data include but are not limited to: risk ratios (RRs) of sleep disorder in the presence of CKD, and the RRs of CKD in the presence of sleep disorder, with corresponding 95% confidence intervals (CI). Where possible, adjusted RRs were used in place of unadjusted RRs.

### Risk of bias assessment and publication bias

Two blinded, independent reviewers (C.Y.J.L. and K.J.M.Y.) conducted the risk of bias assessment of included articles using the Risk of Bias in Non-randomized Studies of Exposures (ROBINS-E) tool. The ROBINS-E is designed specifically to assess the risk of bias of cohort studies on the premises of confounding, measurement of exposure, selection of participants, post-exposure interventions, missing data, measurement of outcome and selection of the reported result [15]. Exclusion of low-quality studies was performed only during sensitivity analyses in an objective to explore the result's heterogeneity. Otherwise, all studies were retained independently of their quality, following Glass's approach [16]. Publication bias was assessed by visual inspection of the respective funnel plots [17]. The asymmetry of funnel plots was further assessed using Egger's linear regression method and Begg's test [18]. Missing studies were imputed using the trim-and-fill method [19].

### Data synthesis and statistical analysis

All analysis was conducted in R Studio (Version 4.2.2) using the *meta* package [20]. Descriptive statistics were presented as means and standard deviations for continuous variables and counts for categorical variables. A conventional pairwise meta-analysis was done in RRs and the results were displayed in forest plots. Statistical heterogeneity was assessed via *I^2^* and Cochran Q test values, where an *I^2^* value of <25% represented low heterogeneity and an *I^2^* value ≥25% represented moderate to high heterogeneity [21]. A Cochran Q test with *P*-value of ≤0.10 was considered significant for heterogeneity. Random effects models were used in all analysis regardless of heterogeneity as recent evidence suggests that it provides more robust outcome measures compared with the alternative fixed effects models [22]. The Hartung–Knapp method was also implemented to adjust the CI of the overall estimate [23].

Where 10 or more studies were available for a particular outcome, additional analyses which were planned *a priori* were conducted to evaluate potential sources of heterogeneity between studies [24]. Apart from subgroup analyses, univariate random-effects meta-regression were conducted, and effect moderators were confirmed using permutation testing with 1000 iterations to eliminate spurious results [25]. Statistical significance was considered for outcomes with a *P*-value ≤.05. Leave-out-one influence analyses were performed to examine the influence of individual studies on the overall findings. Cumulative meta-analyses were performed ranked by year published, to examine the stability of published data over time.

### Certainty of evidence

The quality of pooled evidence was evaluated using the Grading of Recommendations Assessment, Development and Evaluations (GRADE) framework [26]. The GRADE framework rates each study on the basis of study design, consistency, directness, risk of bias, precision and publication bias. For each outcome, the level of evidence was rated as high, moderate, low or very low.

## RESULTS

### Literature search and study characteristics

**Figure 1:**
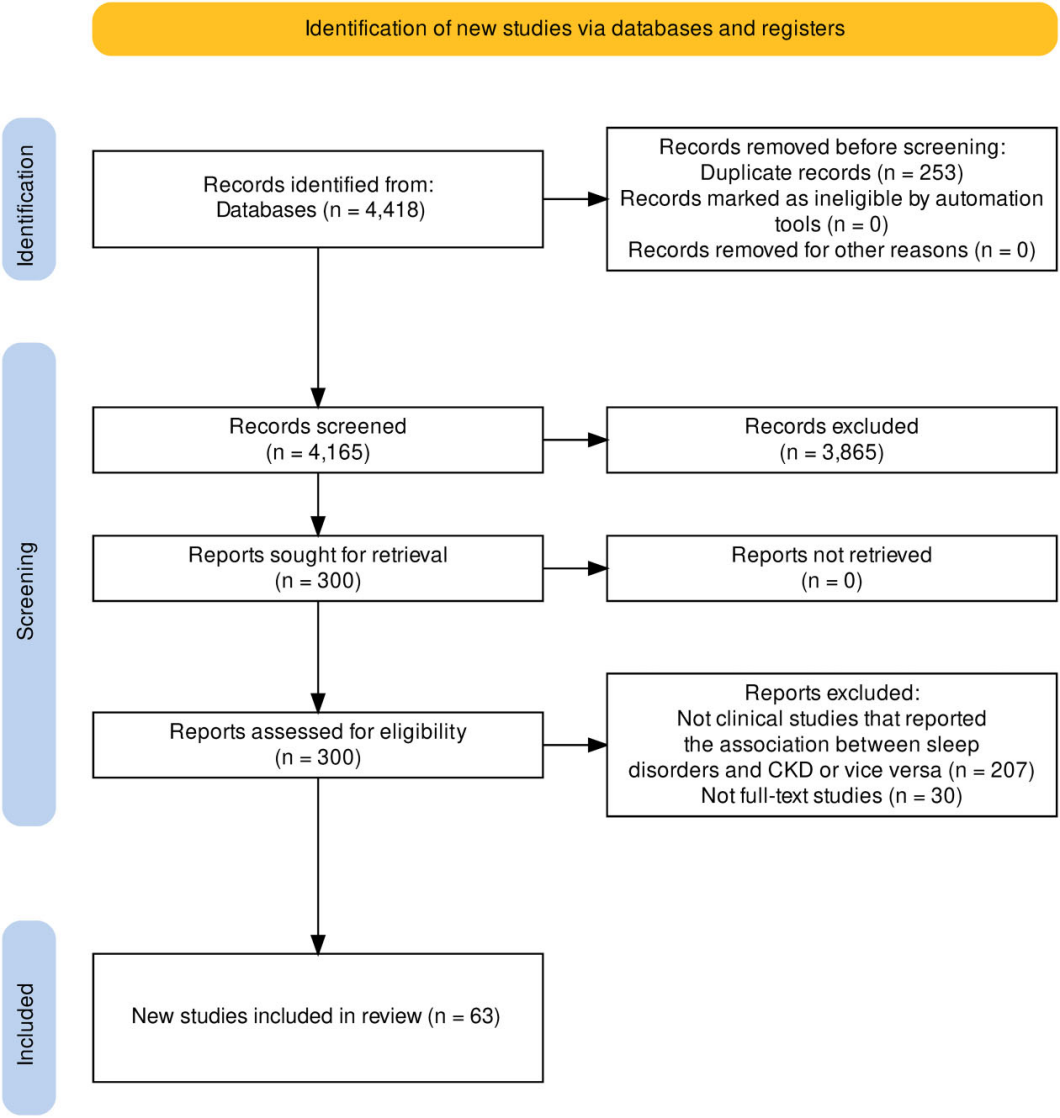
PRISMA flow diagram.

From an initial search of 4418 articles, 3418 articles were included in the initial search after removal of duplicates, of which 300 were selected for full text review. Sixty-three articles met the final inclusion criteria [5, 27–87]. Thirty-four studies were retrospective cohort studies, 3 studies were case–control studies and 26 studies were prospective cohort studies (Fig. 1). The inter-rate reliability as assessed by Cohen's kappa was 0.98 [88].

The total sample size was 26 777 524 participants. The mean age was 58.03 ± 14.21 years, 73% of patients were male and the mean eGFR was 55.34 ± 11.12 mL/min/1.73 m^2^. There were 10 974 271 (41.0%) patients with hypertension, 8 988 735 (33.6%) with hyperlipidemia and 8 957 126 (33.5%) diabetes mellitus. Among patients with CKD, there were 31 616 patients on renal dialysis and 45 patients who received renal transplant (Table 1). Of the 63 included studies, 33 were rated as low risk of bias, 19 as moderate risk of bias and 11 as high risk of bias (Supplement 2).

**Table 1:** Characteristics of included studies.

Author	Study design	Sample size	Mean age (years)	Male (%)	HTN (%)	HLD (%)	DM (%)	CVD (%)	Smoker (%)	Dialysis (%)	Renal transplant (%)
Abdel Rahman, *Geriatr Gerontol Int* (2014) [56]	Cross-sectional study	1200	69.64	100.00	55.5	NR	32.5	23.5	31.3	NR	NR
Abdel-Kader *et al*., *J Hypertens* (2012) [76]	Cohort study	407	56.87	56.27	33.2	NR	26.0	NR	41.5	23.3	0.0
Abdullah *et al*., *Am J Cardiol* (2018) [73]	Cohort study	12 608 637	58.80	43.16	55.1	NR	27.3	35.3	33.4	NR	NR
Adams *et al*., *Sleep* (2017) [38]	Cross-sectional study	1543	61.22	100.00	54.1	NR	19.9	NR	NR	NR	NR
Adderley *et al*., *Diabetes Care* (2020) [65]	Cohort study	34 270	59.14	75.30	55.9	NR	47.0	NR	51.2	NR	NR
Aritake-Okada *et al*., *Sleep Med* (2011) [70]	Cohort study	1049	59.46	68.68	NR	NR	29.0	NR	26.4	NR	NR
Baba *et al*., *Eur J Neurol* (2011) [42]	Cross-sectional study	62	66.00	40.32	NR	NR	31.0	NR	NR	NR	NR
Bambini *et al. Hemodial Int* (2019) [77]	Cross-sectional study	100	47.62	37.00	30.0	NR	40.0	NR	53.1	25.0	0.0
Beecroft *et al*., *Eur Respir J* (2007) [69]	Cross-sectional study	85	47.37	60.00	NR	NR	NR	NR	52.0	51.8	0.0
Bhandari *et al*., *Respirology* (2016) [39]	Cohort study	470 386	65.00	45.00	100.0	NR	32.0	25.0	NR	NR	NR
Brgdar *et al*., *Cureus* (2021) [48]	Cohort study	47 356	65.14	63.80	83.1	58.2	40.6	35.3	NR	NR	NR
Canales *et al*., *Sleep Med* (2008) [72]	Cross-sectional study	508	76.00	NR	NR	NR	NR	NR	NR	NR	NR
Canales *et al*., *Nephrol Dial Transplant* (2008) [89]	Cohort study	2696	72.80	100.00	55.8	NR	8.2	34.3	47.6	0.0	0.0
Chang *et al*., *Medicine (Baltimore)* (2016) [74]	Cross-sectional study	499	50.17	73.10	52.1	20.6	18.0	NR	75.4	NR	NR
Chen *et al*., *J Clin Endocrinol Metab* (2014) [60]	Cohort study	22 032	55.14	82.79	15.5	6.1	8.0	12.5	NR	NR	NR
Chen *et al*., *Sleep Health* (2022) [87]	Cohort study	1657	57.69	45.99	53.7	NR	53.7	NR	NR	NR	NR
Choi *et al*., *Clin Exp Nephrol* (2019) [61]	Cohort study	404 154	53.20	73.10	34.7	NR	13.1	13.8	53.8	NR	NR
Chou *et al*., *Nephrol Dial Transplant* (2011) [68]	Cohort study	40	44.80	83.00	NR	NR	NR	NR	NR	0.0	0.0
Chou *et al*., *Am J Ophthalmol* (2012) [81]	Cohort study	35 634	45.16	62.34	33.9	19.4	17.7	21.9	NR	0.0	0.0
Chu *et al*., *Respirology* (2016) [35]	Cohort study	43 434	46.52	62.80	27.4	NR	14.9	19.0	17.7	0.0	0.0
Ding *et al*., *Int J Environ Res Public Health* (2021) [41]	Cohort study	30 570	NR	69.25	NR	NR	16.6	NR	NR	NR	NR
Dong *et al*., *Endocr J* (2020) [33]	Cross-sectional study	322	56.80	66.67	57.8	57.7	NR	NR	NR	NR	NR
Dou *et al*., *Nephron* (2017) [31]	Cross-sectional study	352	69.00	61.65	79.8	NR	44.8	19.0	NR	1.0	NR
Elias *et al*., *Sleep Med* (2016) [27]	Cross-sectional study	114	52.30	77.10	NR	NR	20.1	NR	NR	100.0	NR
Erridge *et al*., *Obes Surg* (2021) [62]	Case–control study	276 210	61.02	36.47	45.4	10.0	25.6	NR	NR	NR	NR
Full *et al*., *J Am Soc Nephrol* (2020) [66]	Cohort study	1525	62.25	42.70	NR	NR	NR	NR	59.8	NR	NR
Furlan *et al*., *Sleep Med* (2021) [67]	Cross-sectional study	242	63.26	62.80	83.8	NR	44.6	38.1	14.7	0.0	0.0
Furukawa *et al*., *Eur J Endocrinol* (2013) [57]	Cohort study	513	62.04	56.90	47.3	42.8	100.0	8.8	21.1	NR	NR
Giatti *et al*., *Ann Am Thorac Soc* (2021) [37]	Cross-sectional study	1946	49.00	43.40	26.2	54.6	15.7	NR	14.0	NR	NR
Horie *et al*., *Eur J Cardiovasc Nurs* (2021) [46]	Cross-sectional study	1233	64.04	74.94	53.7	44.4	21.3	39.0	59.3	NR	NR
Huang *et al*., *Int J Environ Res Public Health* (2018) [79]	Cohort study	147 805	54.02	46.59	42.7	24.7	15.7	19.0	6.2	20.0	0.0
Huang *et al*., *Medicine (Baltimore)* (2015) [58]	Cohort study	128 436	51.07	35.78	26.9	12.7	4.9	14.1	NR	NR	NR
Hui *et al*., *Ann Palliat Med* (2020) [64]	Cohort study	444	47.72	79.50	48.2	37.2	21.8	NR	NR	NR	NR
Hung *et al*., *BMC Psychiatry* (2018) [78]	Case–control study	310 458	47.39	40.35	14.4	6.5	6.8	5.0	NR	NR	NR
Jackson *et al*., *Thorax* (2021) [55]	Cross-sectional study	1895	68.20	46.28	18.0	33.0	20.0	NR	45.4	NR	NR
Jonsson *et al*., *Clin Kidney J* (2022) [51]	Cohort study	206 727	67.27	43.89	28.1	13.2	6.2	NR	28.1	NR	NR
Jurado-Gamez *et al*., *Nephron Clin Pract* (2007) [85]	Cross-sectional study	51	53.29	70.59	43.1	NR	7.8	NR	25.0	62.7	NR
Kahvecioglu *et al*., *Exp Clin Transplant* (2016) [43]	Cross-sectional study	193	43.73	53.88	34.3	NR	15.1	NR	27.0	60.1	23.3
Kario *et al*., *Circ J* (2016) [49]	Cohort study	535	57.41	60.75	NR	NR	45.0	33.0	14.0	NR	NR
Koo *et al*., *Heart* (2020) [83]	Cohort study	1007	61.63	86.49	74.8	81.2	56.9	100.0	31.7	3.6	0.0
Lee *et al*., *Acta Cardiol Sin* (2017) [90]	Cohort study	193 263	NR	39.68	40.7	28.6	20.8	23.2	NR	NR	NR
Lee *et al*., *Sleep* (2015) [82]	Cohort study	28 044	50.07	66.15	25.0	15.8	10.8	25.1	NR	NR	NR
Leong *et al*., *J Clin Sleep Med* (2014) [47]	Cross-sectional study	90	51.56	43.33	NR	NR	100.0	25.0	NR	NR	NR
Lin *et al*., *Sleep Breath* (2017) [80]	Cohort study	41 196	NR	62.79	30.1	NR	17.4	NR	NR	NR	NR
Linz *et al*., *J Hypertens* (2017) [75]	Cross-sectional study	1868	61.11	58.86	NR	NR	37.6	53.9	9.8	NR	NR
Low *et al*., *Sleep Med* (2013) [53]	Cross-sectional study	160	54.90	89.38	61.3	85.0	35.6	45.7	43.8	NR	NR
Lu *et al*., *Mayo Clin Proc* (2018) [32]	Cohort study	1 639 090	63.20	93.39	57.8	NR	21.6	9.8	NR	NR	NR
Lui *et al*., *Int J Obstet Anesth* (2021) [63]	Cohort study	6 911 916	28.60	0.00	NR	NR	42.0	2.0	3.8	NR	NR
Mehta *et al*., *Bone* (2018) [84]	Case–control study	1204	50.75	65.03	43.1	NR	30.8	7.3	NR	NR	NR
Molnar *et al*., *Thorax* (2015) [34]	Cohort study	3 079 514	60.98	92.97	57.8	NR	57.8	19.0	NR	NR	NR
Nicholl *et al*., *Chest* (2012) [40]	Cross-sectional study	254	59.57	64.57	83.9	NR	37.0	22.0	NR	29.5	0.0
Ogna *et al*., *Sleep* (2016) [5]	Cross-sectional study	6732	56.70	50.68	43.5	NR	10.1	NR	18.5	0.0	0.0
Pinto *et al*., *Sleep Breath* (2016) [28]	Cross-sectional study	39	54.90	NR	NR	NR	39.0	NR	NR	51.3	0.0
Plantinga *et al*., *Am J Kidney Dis* (2011) [36]	Cross-sectional study	9110	47.20	49.80	42.0	NR	8.3	8.5	19.9	0.0	0.0
Quinn *et al*., *Sleep Med* (2011) [54]	Cross-sectional study	301	70.68	47.18	38.5	NR	15.0	29.1	47.2	0.0	0.0
Roumelioti *et al*., *Clin J Am Soc Nephrol* (2011) [86]	Cross-sectional study	388	57.58	NR	NR	NR	26.1	NR	51.1	19.3	0.0
Somay *et al*., *Niger J Clin Pract* (2020) [45]	Cross-sectional study	137	49.09	51.09	NR	NR	31.3	NR	NR	49.6	0.0
Wang *et al*., *BMJ Open* (2021) [30]	Cohort study	32 989	64.18	56.83	44.0	NR	27.8	48.0	NR	4.1	NR
Xu *et al*., *Sleep Med* (2016) [44]	Cohort study	1454	53.73	72.79	27.0	NR	12.0	8.7	9.6	0.0	0.0
Yatsu *et al*., *Clin Res Cardiol* (2018) [52]	Cohort study	539	67.30	84.42	76.3	79.2	43.4	NR	29.8	NR	NR
Yayan *et al*., *Adv Exp Med Biol* (2017) [59]	Cohort study	382	63.75	69.63	68.6	NR	24.6	14.2	21.4	1.3	0.0
Yeo *et al*., *Ann Transl Med* (2017) [50]	Cohort study	42 189	81.10	52.42	81.5	64.3	33.5	71.4	3.0	NR	NR
Yoshihisa *et al*., *Int Heart J* (2019) [71]	Cohort study	338	64.20	73.08	63.9	68.3	45.3	NR	54.4	NR	NR

HTN, hypertension; HLD, hyperlipidemia; DM, diabetes mellitus; CVD, cardiovascular disease; NR, not reported.

### Association of chronic kidney disease in the presence of sleep disorders

Based on the availability of data from the included studies, sleep disorders were classified as OSA, restless leg syndrome (RLS), sleep apnea, insomnia and other sleep disorders. Based on the definitions of sleep apnea from the included studies, sleep apnea was defined as either OSA, central sleep apnea (CSA), mixed sleep apnea or sleep apnea not otherwise specified.

#### Obstructive sleep apnea

**Figure 2:**
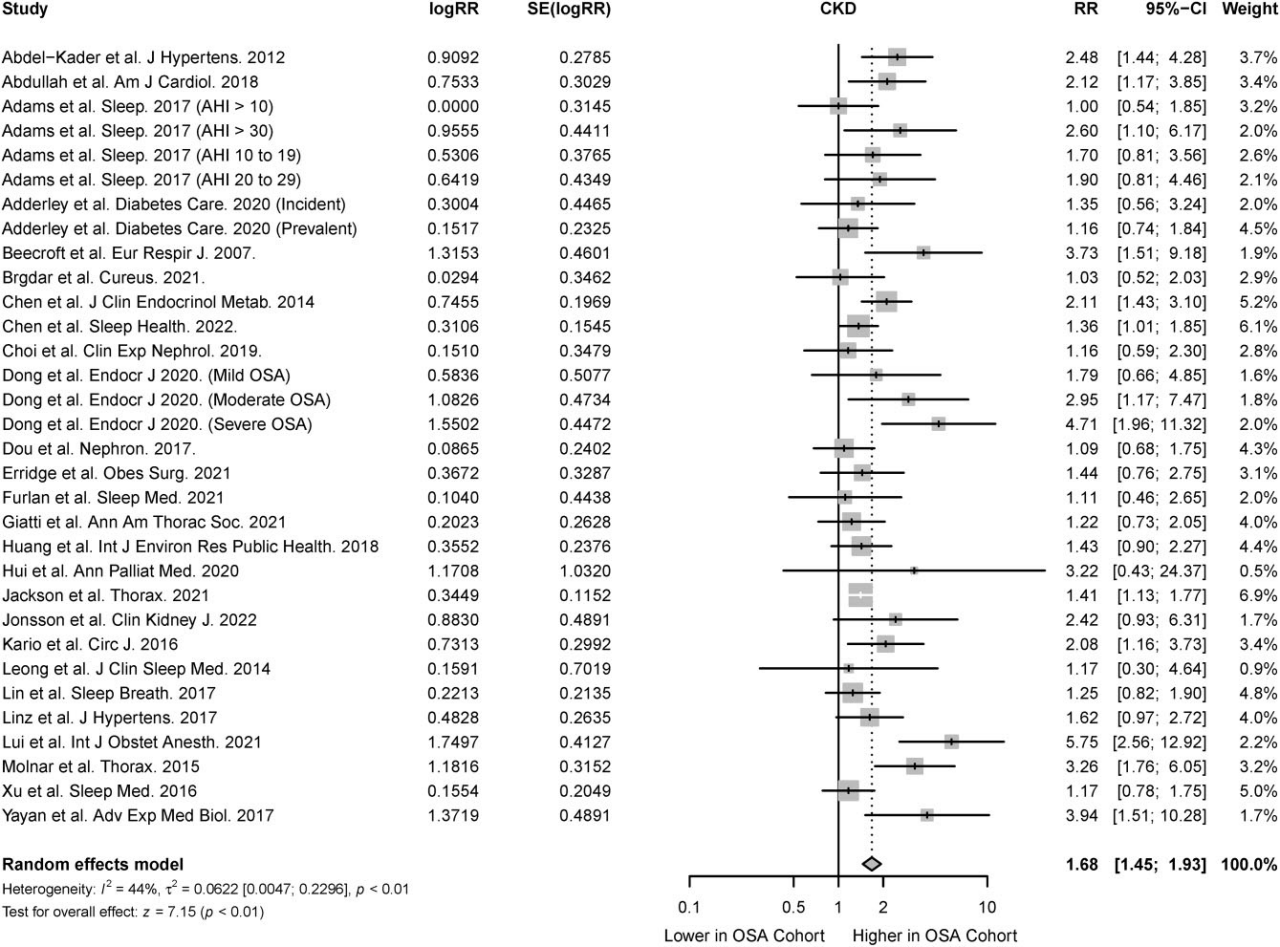
Risk of CKD in the presence of OSA.

**Figure 3:**
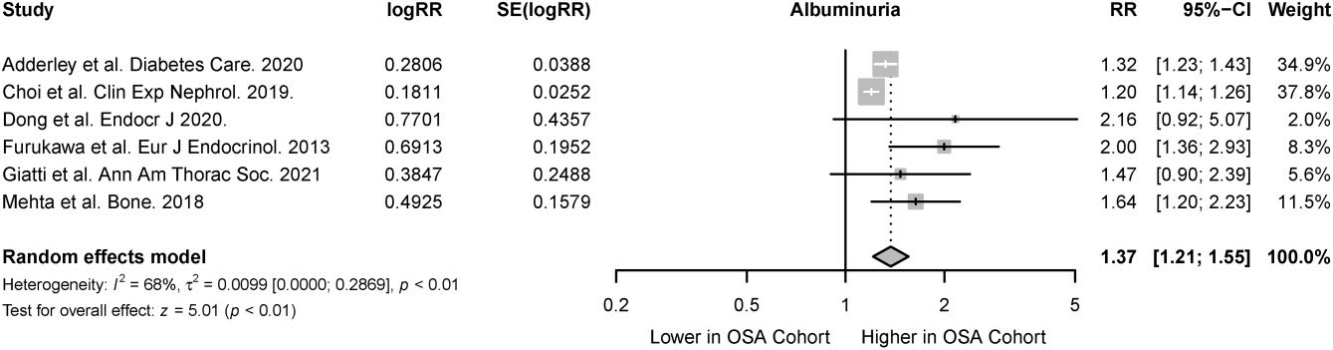
Risk of albuminuria in the presence of OSA.

The association of CKD in the presence of OSA was reported in 26 studies [31, 33, 34, 37, 38, 44, 48, 49, 51, 55, 58–65, 67, 69, 70, 73, 75, 76, 80, 87]. There was a significant association of CKD in patients with OSA (RR 1.68; 95% CI 1.45 to 1.93; *I^2^* = 44%; *P* < .01) (Fig. 2), both among cohort studies which investigated the risk of incident CKD in OSA patients (RR 1.59; 95% CI 1.25 to 2.02; *I^2^* = 46; *P* < .01) and cross-sectional studies that investigated the association between OSA and prevalent CKD (RR 1.74, 95% CI 1.48 to 2.05; *I^2^* = 49; *P* < .01) (Table 2). From six studies, the presence of albuminuria was also significantly higher in patients with OSA compared with patients without OSA (RR 1.37, 95% CI 1.21 to 1.55; *I^2^* = 68; *P* < .01) (Fig. 3) [33, 37, 57, 61, 65, 84].

**Table 2:** Results of the subgroup analysis.

Outcome	Studies	RR (95% CI)	95% PI	*I* ^2^ (%)	*P*-value for subgroup differences
Risk of CKD in the presence of OSA					
Study design					
Prospective cohort studies (incident CKD)	7	1.59 (1.25–2.02)	0.84–3.43	46	.54
Cross-sectional studies (prevalent CKD)	20	1.74 (1.48–2.05)	0.93–3.35	49	
Geographical region					
Americas	11	2.25 (1.64–3.08)	0.81–5.04	63	.15
Europe	5	1.56 (1.10–2.23)	0.74–3.29	20	
Asia-Pacific	7	1.64 (1.34–2.00)	1.26–2.13	0	
Method of eGFR calculation					
MDRD	2	2.24 (1.35–3.72)	NA	0	.14
CKD-EPI	9	1.46 (1.13–1.89)	0.77–2.78	39	
Stage of CKD					
3–5	10	1.51 (1.25–1.81)	0.96–0.36	35	.57
1–5	13	1.76 (1.40–2.21)	0.93–3.43	42	
5 only	3	1.63 (0.88–3.02)	0.88–3.02	43	
RRT					
Included patients on RRT	6	1.76 (1.23–2.52)	0.71–4.35	38	.69
Does not included patients on RRT	21	1.62 (1.40–1.88)	0.97–2.72	47	
Method of diagnosis of OSA					
PSG	5	1.69 (1.47–1.95)	1.02–2.62	41	.79
ICD-9 codes	22	1.80 (1.18–2.73)	0.45–7.20	71	
					
Risk of CKD in the presence of sleep apnea					
Study design					
Prospective cohort studies (incident CKD)	4	1.94 (1.39–1.98)	1.18–3.20	38	.43
Cross-sectional studies (prevalent CKD)	14	1.57 (1.35–1.85)	1.01–2.45	36	
Geographical region					
Americas	9	1.46 (1.20–1.78)	0.96–2.23	25	.38
Asia	8	1.83 (1.36–2.45)	0.70–4.74	79	
Europe	1	1.89 (1.08–3.33)	NA	NA	
Method of eGFR calculation					
MDRD	3	1.38 (1.05–1.82)	0.15–2.85	36	.09
CKD-EPI	5	1.98 (1.46–2.68)	0.69–2.77	49	
Stage of CKD					
3–5	8	1.57 (1.17–2.1)	0.82–3.23	59	.69
5 only	2	1.70 (1.06–2.71)	NA	0	
1–5	8	1.80 (1.2–2.7)	0.66–4.35	81	
RRT					
Included patients on RRT	6	1.68 (1.29–2.19)	0.90–3.14	31	.87
Does not included patients on RRT	12	1.64 (1.31–2.05)	0.75–3.57	72	
Method of diagnosis of OSA					
PSG	12	1.59 (1.29–1.95)	0.83–3.03	55	.50
ICD-9 codes	6	1.80 (1.32–2.45)	0.69–4.72	73	
Risk of OSA in the presence of CKD					
Study design					
Prospective cohort studies (incident OSA)	8	1.60 (1.35–1.89)	1.30–1.97	0	.32
Cross-sectional studies (prevalent OSA)	20	1.79 (1.55–2.07)	1.11–3.01	41	
Geographical region					
Americas	11	1.75 (1.38–2.22)	1.03–3.10	23	.76
Europe	5	1.64 (1.24–2.15)	1.11–2.41	0	
Asia-Pacific	12	1.61 (1.43–1.82)	1.41–1.88	0	
Method of eGFR calculation					
MDRD	3	1.84 (1.19–2.84)	0.06–5.33	16	.32
CKD-EPI	8	1.46 (1.25–1.70)	1.21–1.77	0	
Stage of CKD					
5	3	1.90 (1.37–2.64)	0.21–1.12	0	.63
3–5	10	1.66 (1.25–2.22)	0.70–3.93	62	
1–5	15	1.85 (1.60–2.14)	1.56–2.09	0	
RRT					
Included patients on RRT	5	1.72 (1.24–2.38)	1.77–3.80	26	.86
Does not included patients on RRT	23	1.77 (1.55–2.03)	1.14–2.74	39	
Method of diagnosis of OSA					
PSG	23	1.62 (1.47–1.79)	1.47–1.83	0	.13
ICD-9 codes	5	2.15 (1.52–3.04)	0.73–6.30	63	
Risk of sleep apnea in the presence of CKD					
Study design					
Prospective cohort studies (incident sleep apnea)	4	1.74 (1.21–2.50)	0.66–4.61	12	.36
Cross-sectional studies (prevalent sleep apnea)	16	1.45 (1.24–1.7)	0.81–2.62	62	
Geographical region					
North America	8	1.40 (1.14–1.73)	0.74–2.47	63	.13
Asia	11	1.85 (1.55–2.21)	1.48–2.31	0	
Europe	1	1.89 (0.33–10.79)	NA	NA	
Method of eGFR calculation					
MDRD	5	1.61 (1.29–2.01)	0.83–3.12	51	.73
CKD-EPI	5	1.30 (0.87–1.96)	0.35–4.89	75	
Cockroft–Gault	2	1.78 (1.15–2.75)	1.15–2.75	12	
Mayo Clinic	2	1.38 (0.68–2.82)	0.68–2.82	79	
Stage of CKD					
3–5	10	1.35 (0.97–1.9)	0.65–3.35	75	.48
1–5	9	1.55 (1.27–1.89)	1.14–2.03	24	
5	2	0.90 (0.42–1.93)	0.42–1.93	0	
RRT					
Included patients on RRT	8	1.57 (1.22–2.01)	0.83–2.96	46	.62
Does not included patients on RRT	12	1.45 (1.22–2.01)	0.76–2.77	64	
Method of diagnosis of OSA					
PSG	14	1.46 (1.21–1.76)	0.72–2.96	65	.91
ICD-9 codes	5	1.48 (1.24–1.77)	1.20–3.26	14	

CKD-EPI, CKD Epidemiology Collaboration; ICD-9, International Classification of Diseases, 9th edition; MDRD, Modification of Diet in Renal Disease; PSG, polysomnography; NA, not applicable.

Meta-regression found that the percentage of patients with end -stage renal disease (ESRD) and year of study completion were statistically significant effect moderators of the association between OSA and CKD (Supplement 4). The percentage of patients with ESRD accounted for 76.80% of heterogeneity and 14.26% of residual heterogeneity, with the pooled RR increasing by a factor of 0.9112 (95% CI 0.0472 to 1.7752) per 1% increase in the number of patients with ESRD. Year of study completion accounted for 23.58% of heterogeneity and 39.87% of residual heterogeneity, with the pooled RR decreasing by a factor of 0.0482 (95% CI –0.0921 to –0.0440) per 1-year increase in the year of study completion. Other covariates including percentage of patients on hemodialysis, peritoneal dialysis and renal transplant were not significant effect moderators of the association between OSA and CKD.

In subgroup analyses (Table 2), the pooled association was significant among studies that included patients with CKD stage 3–5 (RR 1.51; 95% CI 1.25 to 1.81; *I^2^* = 35) and 1–5 (RR 1.76; 95% CI 1.40 to 2.21; *I^2^* = 42), but not among studies that included patients with CKD stage 5 only (RR 1.63; 95% CI 0.88 to 3.02; *I^2^* = 43). The pooled association also remained significant among studies that included patients on RRT (RR 1.76; 95% CI 1.23 to 2.52; *I^2^* = 38) and studies that did not (RR 1.62; 95% CI 1.40 to 1.88; *I^2^* = 47). The pooled association otherwise remained significant and similar across all subgroups of geographical region, method of eGFR calculation and the modality of diagnosis of OSA (Table 2).

#### Sleep apnea

**Figure 4:**
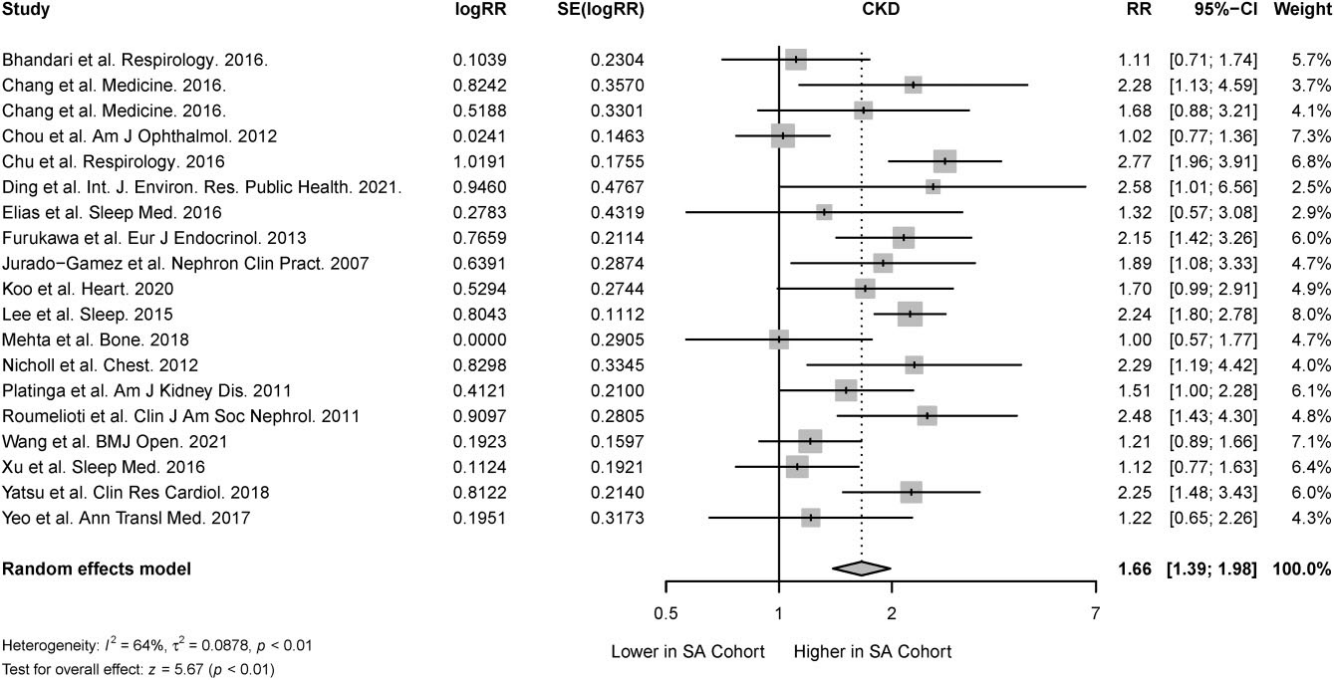
Risk of CKD in the presence of sleep apnea.

From 18 studies that reported the association of CKD in the presence of sleep apnea [27, 30, 35, 36, 39–41, 43, 44, 50, 52, 74, 81–85, 86], there was a significant association between sleep apnea and CKD (RR 1.66; 95% CI 1.39 to 1.98; *I*^2^ = 64%; *P* < .01) (Fig. 4). Among cohort studies which investigated the risk of incident CKD in sleep apnea patients, there was a significant association between sleep apnea and incident CKD (RR 1.94; 95% CI 1.39 to 1.98; *I^2^* = 38; *P* < .01). There was also a significant association between sleep apnea and prevalent CKD in cross-sectional studies (RR 1.57; 95% CI 1.35 to 1.85; *I^2^* = 36; *P* < .01) (Table 2). The percentage of patients with ESRD or on hemodialysis, peritoneal dialysis and renal transplant were not significant effect moderators of the association between sleep apnea and CKD.

In subgroup analyses (Table 2), the pooled association was significant among studies that included patients with CKD stage 3–5 (RR 1.57; 95% CI 1.17 to 2.10; *I^2^* = 59), CKD stage 1–5 (RR 1.80; 95% CI 1.20 to 2.70; *I^2^* = 81) and CKD stage 5 only (RR 1.70; 95% CI 1.06 to 2.71; *I^2^* = 0). The pooled association also remained significant among studies that included patients on RRT (RR 1.76; 95% CI 1.23 to 2.52; *I^2^* = 38) and studies that did not (RR 1.64; 95% CI 1.31 to 2.05; *I^2^* = 72). There were no significant differences in the RR between subgroups.

#### Restless legs syndrome, insomnia and other sleep disorders

**Figure 5:**
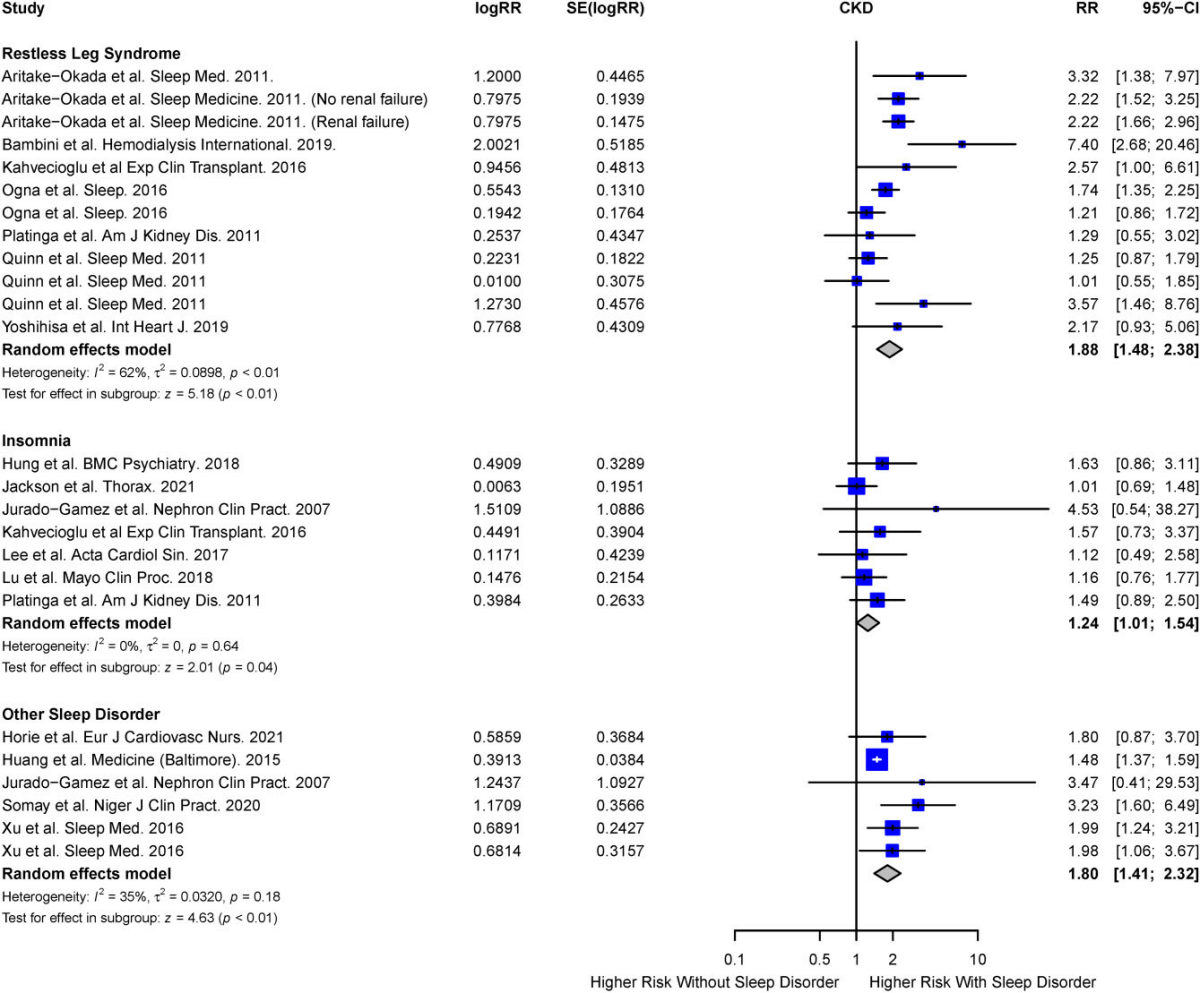
Risk of CKD in the presence of RLS, insomnia and other sleep disorders.

The risk of CKD in the presence of RLS, insomnia and other sleep disorders were reported in eight studies each for RLS [5, 36, 43, 54, 70, 71, 77, 85] and insomnia [32, 36, 43, 55, 78, 82, 85], and five studies [44–46, 58, 85] for other sleep disorders. Other sleep disorders reported include excessive daytime sleepiness, frequent nightmares, nocturia and sleep bruxism, which did not fall into the categories of sleep disorders described above. Based on the random-effects model, a significantly increased risk of CKD was observed in the RLS cohort (RR 1.88; 95% CI 1.48 to 2.38; *I^2^* = 52%; *P* < .01), insomnia cohort (RR 1.24; 95% CI 1.01 to 1.54; *I^2^* = 0%; *P* = .04) and other sleep disorder cohort (RR 1.80; 95% CI 1.41 to 2.32; *P* < .01) (Fig. 5).

### Association of sleep disorders in the presence of chronic kidney disease

#### Obstructive sleep apnea

**Figure 6:**
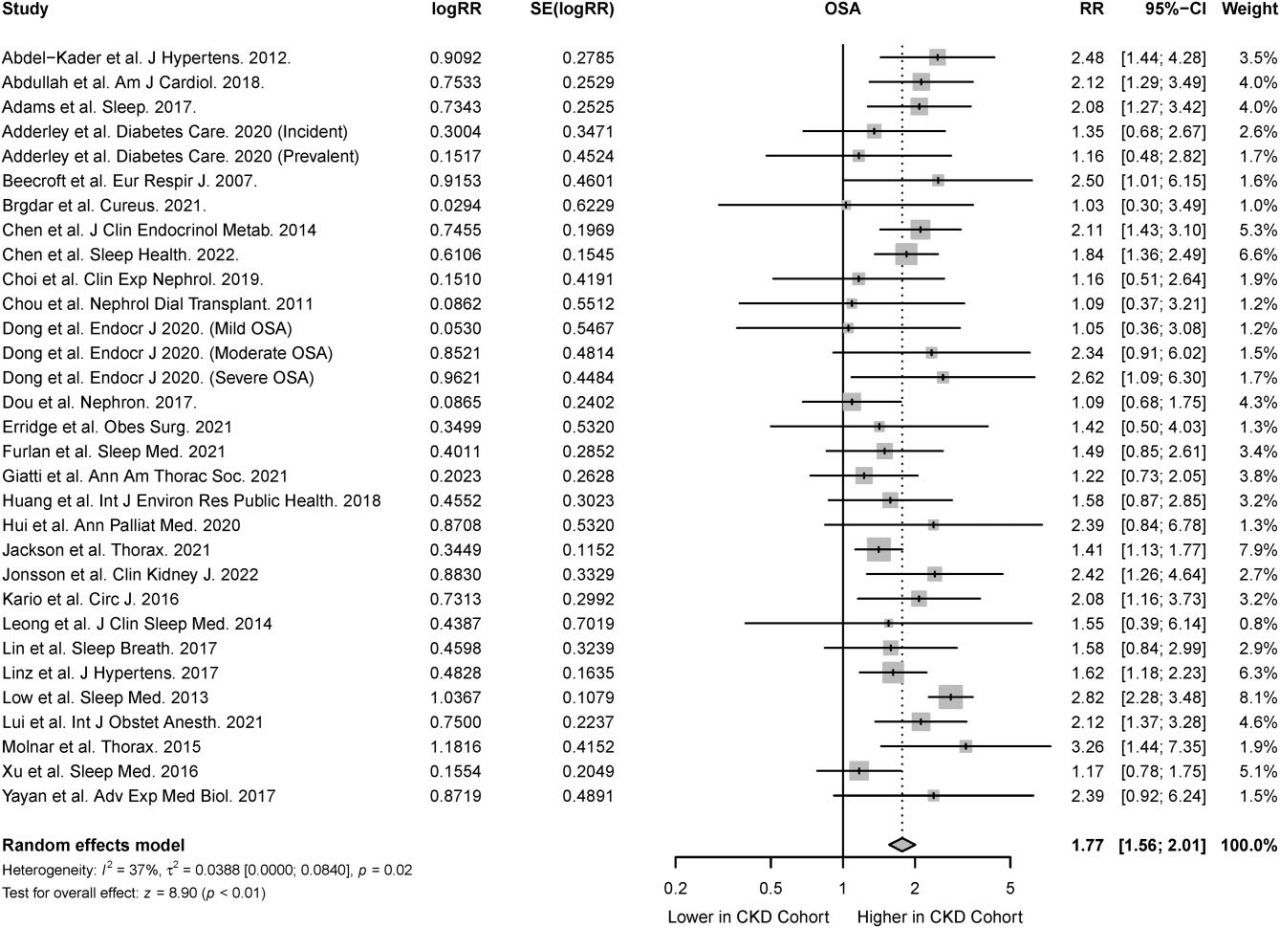
Risk of OSA in the presence of CKD.

**Figure 7:**
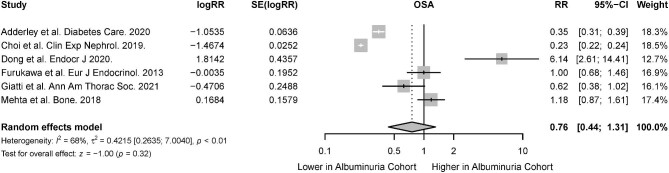
Risk of OSA in the presence of albuminuria.

The association of OSA with CKD was reported in 29 studies [31, 33, 34, 37, 38, 44, 47–49, 51, 53, 55, 58–65, 67–70, 73, 75, 76, 80, 87]. Random-effects meta-analysis demonstrated that the presence of CKD was associated with a significantly higher risk of OSA (RR 1.77; 95% CI 1.56 to 2.01; *I^2^* = 37%; *P* < .01) (Fig. 6). Among cohort studies which investigated the risk of incident OSA in CKD patients, there was a significant association between CKD and incident OSA (RR 1.60; 95% CI 1.35 to 1.89; *I^2^* = 0; *P* < .01). There was also a significant association between CKD and prevalent OSA in cross-sectional studies (RR 1.79; 95% CI 1.55 to 2.07; *I^2^* = 41; *P* < .01) (Table 2). The risk of OSA was not significantly higher in patients with albuminuria compared with patients without albuminuria (RR 0.76; 95% CI 0.44 to 1.31; *I^2^* = 68; *P* = .32) (Fig. 7) [33, 37, 57, 61, 65, 84].

In subgroup analyses (Table 2), the pooled association was significant among studies that included patients with CKD stage 3–5 (RR 1.66; 95% CI 1.25 to 2.22; *I^2^* = 62), CKD stage 1–5 (RR 1.85; 95% CI 1.60 to 2.14; *I^2^* = 0) and CKD stage 5 only (RR 1.90; 95% CI 1.37 to 2.64; *I^2^* = 0). The pooled association also remained significant among studies that included patients on RRT (RR 1.72; 95% CI 1.24 to 2.38; *I^2^* = 26) and studies that did not (RR 1.77; 95% CI 1.55 to 2.03; *I^2^* = 39). There were no significant differences in the RR between subgroups.

Meta-regression found that year of study completion was a statistically significant effect moderator of the odds ratio for developing OSA, accounting for 73.23% of heterogeneity with low (11.36%) residual heterogeneity. The pooled RR decreased by a factor of 0.0412 (95% CI –0.0704 to –0.120) per 1-year increase in year of study completion (Supplement 4). Other covariates including percentage of patients with ESRD or on hemodialysis, peritoneal dialysis and renal transplant were not significant effect moderators of the association between OSA and CKD.

#### Sleep apnea

**Figure 8:**
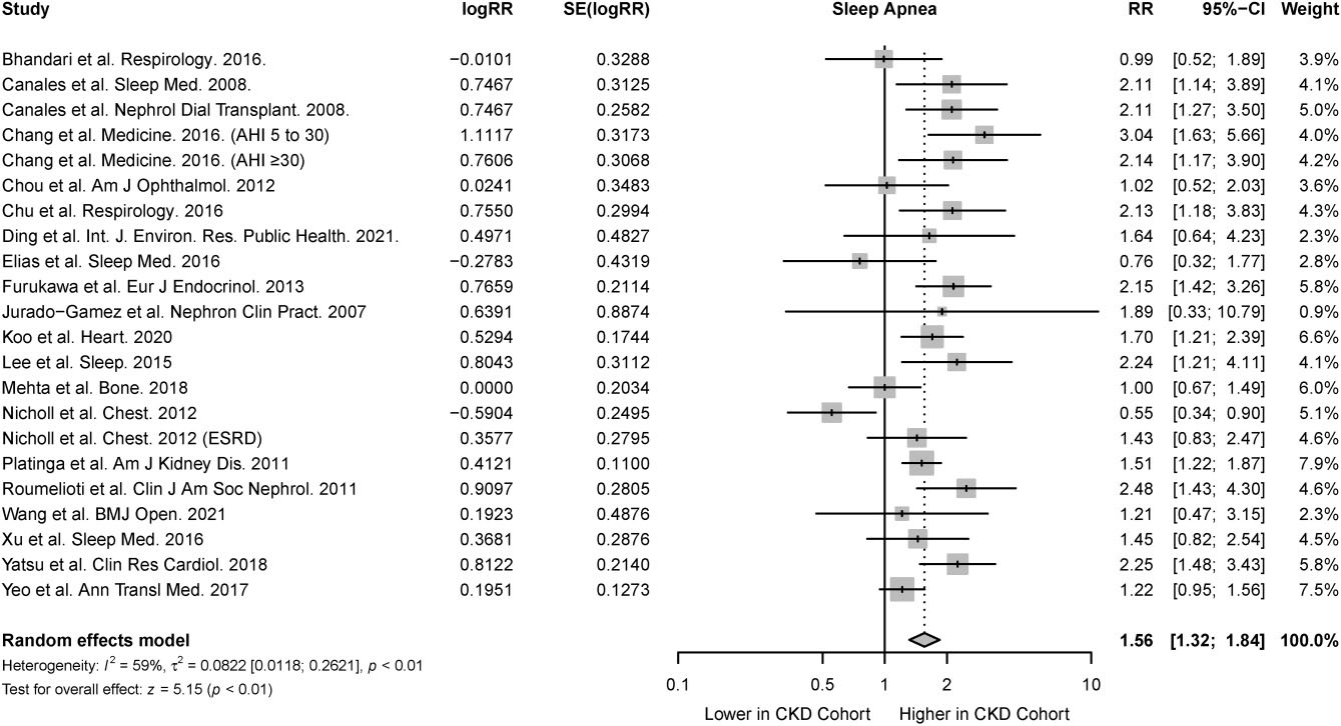
Risk of sleep apnea in the presence of CKD.

The association of CKD with sleep apnea was reported in 20 studies [27, 30, 35–36, 39–41, 44, 50, 52, 57, 72, 74, 81–86, 89]. Based on the random-effects model, the presence of sleep apnea was associated with a significantly increased risk of CKD (RR 1.56; 95% CI 1.32 to 1.84; *I^2^* = 59%; *P* < .01) (Fig. 8). Among studies which investigated the risk of incident sleep apnea in CKD patients, there was a significant association between CKD and incident sleep apnea (RR 1.74; 95% CI 1.21 to 2.50; *I^2^* = 12; *P* = .03). There was also a significant association between CKD and prevalent sleep apnea in cross-sectional studies (RR 1.45; 95% CI 1.24 to 1.70; *I^2^* = 62; *P* = .02) (Table 2).

Meta-regression found that percentage of smokers and percentage of patients on renal transplant were statistically significant effect moderators of the RR for developing sleep apnea, accounting for 100.00% and 19.86% of heterogeneity respectively. The pooled RR increased by a factor of 1.0727 (95% CI 0. 4487 to 1.6967) per 1% increase in the number of smokers, and the pooled RR increased by a factor of 0.3364 (95% CI –0.5621 to –0.1744) per 1% increase in the number of patients who underwent renal transplant (Supplement 4). Other covariates including percentage of patients with ESRD or on hemodialysis and peritoneal dialysis were not significant effect moderators of the association between sleep apnea and CKD.

In subgroup analyses, the pooled association was significant among studies that included patients with CKD stage 1–5 (RR 1.55; 95% CI 1.27 to 1.89; *I^2^* = 24), but not among studies that included patients with CKD stage 5 only (RR 0.90; 95% CI 0.42 to 1.93; *I^2^* = 0) and CKD stage 3–5 (RR 1.35; 95% CI 0.97 to 1.90; *I^2^* = 75). The pooled association also remained significant among studies that included patients on RRT (RR 1.57; 95% CI 1.22 to 2.01; *I^2^* = 46) and studies that did not (RR 1.45; 95% CI 1.22 to 2.01; *I^2^* = 64). There were no significant differences in the RR between subgroups.

#### Restless legs syndrome, insomnia and other sleep disorders

**Figure 9:**
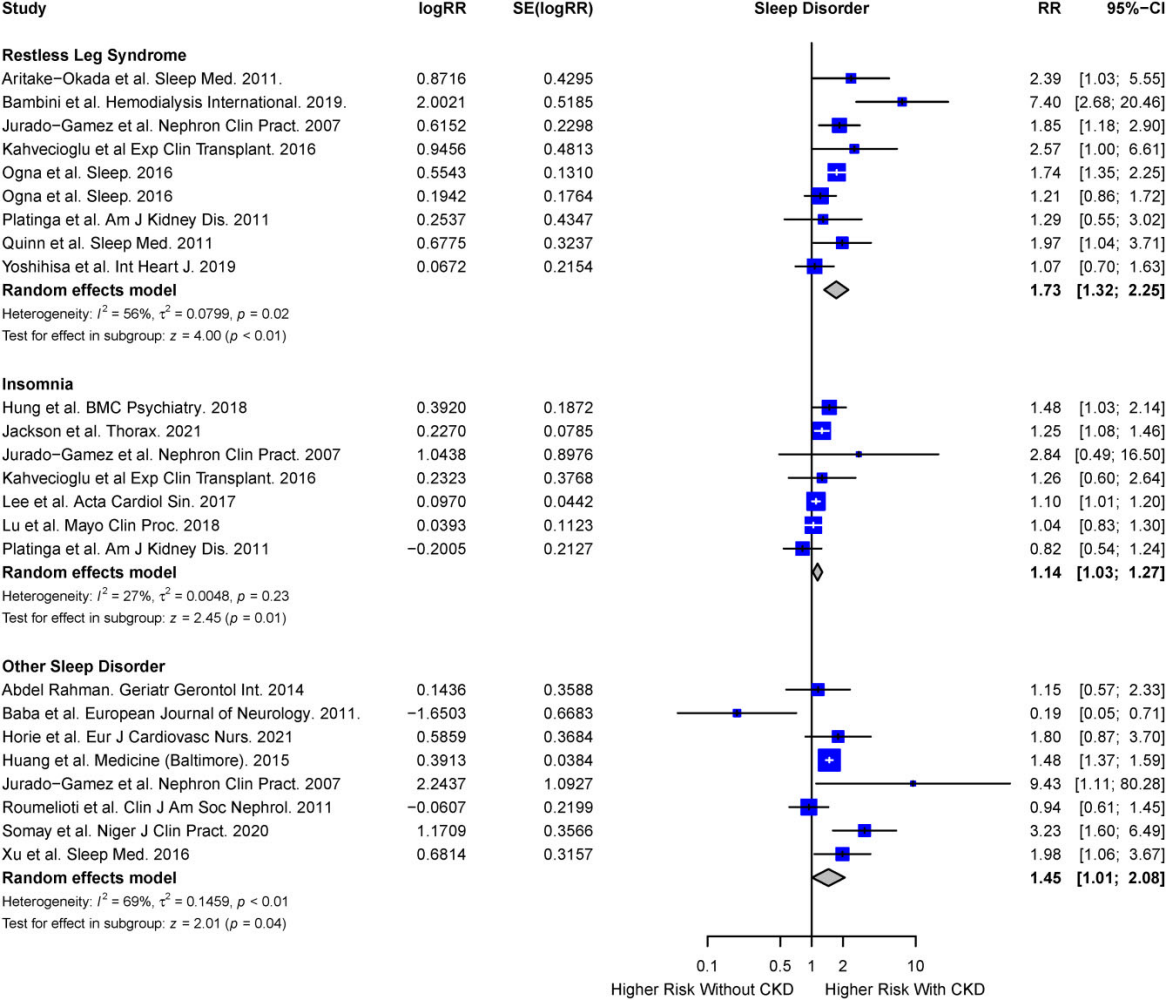
Risk of RLS, insomnia and other sleep disorders in presence of CKD.

The risk of RLS, insomnia and other sleep disorders in the presence of CKD was reported in eight [5, 36, 43, 54, 70, 71, 77, 85], seven, [32, 36, 43, 55, 78, 82, 85] and six studies [44–46, 56, 58, 85], respectively. Other sleep disorders reported include excessive daytime sleepiness, frequent nightmares, nocturia and sleep bruxism, which did not fall into the categories of sleep disorders described above. Based on the random-effects model, a significantly increased risk of RLS (RR 1.73; 95% CI 1.32 to 2.25; *I^2^* = 56%; *P* < .01), insomnia (RR 1.14; 95% CI 1.03 to 1.27; *I^2^* = 27%; *P* = .01) and other sleep disorders (RR 1.90; 95% CI 1.39 to 2.61; *I^2^* = 48%; *P* < .01) were observed in the CKD cohort (Fig. 9).

### Publication bias

For all outcomes, while visual inspection suggested the possibility of funnel plot asymmetry, this was not shown by Egger's test. Trim-and-fill resulted in minimal change to the pooled effect sizes. For all analyses, leave-one-out influence analysis showed that no single study had a drastic change on the pooled RR, and cumulative meta-analysis showed a significant and stable pooled effect size (Supplement 5).

### GRADE quality of evidence

The certainty of evidence for the associations of sleep disorder with CKD were assessed using the GRADE framework. The certainty of evidence for association of CKD in the presence of OSA (moderate), sleep apnea (moderate), RLS (low), insomnia (moderate) and other sleep disorder (moderate) is shown in Supplement 6. The certainty of evidence for association of OSA (moderate), sleep apnea (moderate), RLS (low), insomnia (low) and other sleep disorders (low) in the presence of CKD is also shown in Supplement 6.

## DISCUSSION

In this systematic review and meta-analysis of 63 studies with a pooled cohort of 26 777 524 participants, we observed that a bidirectional association was observed between obstructive sleep apnea, RLS, sleep apnea and CKD [5, 27–86, 87, 90]. The presence of albuminuria was also significantly higher in patients with OSA compared with patients without OSA. Meta-regression found that year of study completion was a significant effect modifier for the bidirectional associations between OSA and CKD. The percentage of smokers was also a significant effect modifier of the risk of sleep apnea in patients with CKD. Insomnia was also found to increase the risk of CKD. Subgroup analysis found that the observed bidirectional association of OSA with CKD remained consistent across geographical regions, eGFR formula and mode of diagnosis of sleep disorder. This meta-analysis provides a comprehensive update on the bidirectional association between CKD and albuminuria with sleep disorders, with further stratification of patients based on the stage of CKD and presence of RRT. We also included pooled estimates on the association of CKD with other sleep disorders such as insomnia and RLS. Given that OSA is a potentially modifiable risk factor, early identification of sleep disorders may allow for early interventions to minimize treatment delays and improve outcomes in terms of renal function. Additionally, early treatment of sleep disorders in patients with CKD may also have positive effects to their quality of life.

The bidirectional association of OSA with CKD is in agreement with the chronic hypoxia hypothesis for the pathogenesis of CKD, first proposed in 1998 [91]. Since then, both human and *in vitro* studies have demonstrated a causal relationship between hypoxia and CKD. The mechanism of this is postulated to be due to inflammation, microvascular insufficiency with glomerular injury, tubulointerstitial fibrosis and consequently decline in renal function [92]. Given that OSA is characterized by intermittent nocturnal hypoxia, this supports the hypothesis of an association between OSA and CKD. Furthermore, OSA is characterized by a systemic chronic inflammatory state, which may itself predispose to renal injury [93]. While OSA may increase the risk of progression of pre-existing CKD and increase the risk of developing *de novo* renal damage, CKD may contribute to the development of OSA and complicate the pre-existing course of OSA. It was interesting to note that meta-regression did not find the percentage of patients with hypertension, hyperlipidemia or diabetes mellitus as a significant effect moderator of the bidirectional association of OSA and CKD. Similarly, the meta-regression also found that mean eGFR was not a significant effect moderator of this bidirectional association. This suggests that the association between OSA and CKD may be seen across different stages and severities of CKD. Nonetheless, due to the design of the study, it was difficult to evaluate the impact of a number of comorbidities, for example hypertension, considering the absence of data on clinical data on these comorbidities. OSA may also increase aldosterone levels, which can lead or increase kidney damage, and partially explain the efficacy of mineralocorticoid receptor antagonists in such patients. Further studies may therefore be useful in confirming if the association between OSA and CKD is indeed independent of these comorbidities.

Apart from obstructive sleep apnea, this study also found a significant bidirectional association between sleep apnea and CKD. For the purposes of this analysis and within the availability of published evidence, sleep apnea was defined as a combination of CSA, OSA or mixed sleep apnea. These findings suggest that other forms of sleep apnea may also contribute to the development of kidney injury. Similar results have been demonstrated in the literature, showing that the mechanisms underlying sleep apnea and CKD may be similar to those implicated in OSA and CKD. These mechanisms can include increased chemosensitivity, hypervolemia, fluid shifts and uremia-induced myopathy [94]. The altered chemoreflex responsiveness and subsequent ventilatory instability may lead to destabilization of respiratory control and periodic breathing, therefore contributing to the pathogenesis of CSA [95]. Mixed sleep apnea is characterized by features of both OSA and CSA, hence patients with mixed sleep apnea may be of similar risk of CKD as patients with either OSA or CSA alone. While data regarding the risk of CKD in patients with CSA alone was not available, further epidemiological studies may be useful in confirming this apparent relationship between CSA and CKD.

Apart from sleep apnea, the meta-analysis demonstrated a bidirectional relationship between RLS and insomnia with CKD. Similar results have also been reported in the literature pertaining to CKD being a risk factor for sleep disorders. Up to 45% of patients with CKD have been reported to experience insomnia, compared with 18% in healthy controls [12]. Insomnia frequently occurs with cardiovascular and psychiatric disease, which further complicates the clinical course of CKD [96]. Published data has also demonstrated shown that the prevalence of sleep disturbances increase with the stage and severity levels of CKD. The progression could lead to a vicious cycle of sleep disorders, CKD and complications of this disease. Similarly, RLS has been found to be present in 20%–57% of CKD patients, with an increased prevalence being noted among patients receiving hemodialysis or peritoneal dialysis [97]. Recent evidence suggests that RLS is both underrecognized and underdiagnosed in renal disease, despite the association between RLS and CKD having been widely described [98]. CKD may increase the risk of RLS through the buildup of uremic waste products, while RLS may decrease the quality of sleep and lead to sleep fragmentation, which may itself be contributory to the pathogenesis of CKD [12]. The results of this meta-analysis highlight the correlates of insomnia and RLS with CKD, and further high quality observational studies may be warranted in confirming these apparent relationships.

The strengths of this study lie in the large number of systematically included studies from diverse settings. Through a rigorous prespecified protocol of systematic searching, bias assessment and quality grading according to international guidelines, these help to enhance the generalizability of these findings. Of the 63 included studies, only 11 studies were found to be of high risk of bias, and their exclusion did not change the findings to of the meta-analysis significantly. Meta-regression was able to adequately explain the observed heterogeneity in the meta-analysis, and the pooled effect sizes were robust to subgroup analyses, meta-regression, influence, cumulative and small-study analyses. Overall, minimal evidence of publication bias was found.

There were appreciable limitations of this meta-analysis. First, the observational nature of the included studies does not permit causal conclusion due to the inability to exclude residual confounding. Second, it was not possible to examine the pooled effect sizes of the association of CKD with CSA or mixed sleep apnea alone due to insufficient stratification of data within the included studies. Nonetheless, the authors believe that the lack of available data reflects the paucity of literature on this topic presently, having performed a comprehensive search of the literature. Third, the conduct of meta-regression was limited by the availability of published literature and could only be performed among studies which reported data on the particular covariate. Aggregate or ecological bias could not be excluded, due to the assumption of linearity. Fourth, a number of included studies in the meta-analysis did not report the number of patients with comorbidities such as hypertension, hyperlipidemia and diabetes, which limited the strength of conclusions that could be drawn from the meta-regression analysis.

## CONCLUSION

In this systematic review and meta-analysis of 63 studies involving 26 868 391 participants, a bidirectional association was observed between CKD and obstructive sleep apnea, RLS and insomnia. Meta-regression revealed that mean age, gender and percentage of patients with hypertension, hyperlipidemia and diabetes did not significantly alter the pooled effect sizes. This study highlights the relationships between sleep disorders and CKD and adds to the growing evidence base suggesting the coexistence of both disorders. Physicians treating patients with sleep disorders should be aware of this association with CKD and vice versa, and adopt measures targeted at addressing the co-existence of these disorders.

## Supplementary Material

sfae279_Supplemental_Files

## Data Availability

All articles in this manuscript are available from PubMed/Medline, Embase, Cochrane Library and CINAHL.
