# Supplementary material for: Bidirectional association of sleep disorders with chronic kidney disease: a systematic review and meta-analysis
Source: Clin Kidney J. 2024 Oct 18;17(11):sfae279. doi: 10.1093/ckj/sfae279 (PMC11549560; doi:10.1093/ckj/sfae279)
Supplement: sfae279_Supplemental_Files [file sfae279_supplemental_files.zip › S1. Search Strategy.docx]

**Supplement 1.** Search Strategy

*Search strategy for Medline*

1. exp kidney failure, chronic/ or (((kidney or renal) and (disease or failure) and (end-stage or end stage or chronic)) or ESRD).tw.

2. exp kidney transplantation/ or (((renal or kidney) and transplant*) or (kidney and grafting)).tw.

3. exp proteinuria/ or (proteinuria* or albuminuria*).tw.

4. exp renal insufficiency, chronic/ or (chronic and (kidney or renal) and (insufficienc* or disease*)).tw.

5. exp sleep apnea, obstructive/ or ((sleep and (apnea or apnoea)) or OSAHS).tw.

6. exp Sleep Initiation and Maintenance Disorders/ or (DIMS or (Disorders of Initiating and Maintaining Sleep) or insomnia* or sleeplessness or (sleep and dysfunction*) or (early and awakening)).tw.

7. 1 or 2 or 3 or 4

8. 5 or 6

9. 7 and 8

*Search strategy for Embase*

1. 'sleep disordered breathing'/exp OR 'sleep disordered breathing' OR ((nocturnal:ti,ab,kw OR sleep:ti,ab,kw) AND (apnea:ti,ab,kw OR apnoea:ti,ab,kw)) OR 'sleep-disordered breathing':ti,ab,kw OR 'hypopnea syndrome':ti,ab,kw OR 'hypopnoea syndrome':ti,ab,kw

2. 'sleep disorder'/exp OR 'insomnia'/exp OR (sleep:ti,ab,kw AND (disorder*:ti,ab,kw OR disturbance*:ti,ab,kw)) OR 'insomnia':ti,ab,kw OR agrypnia:ti,ab,kw OR hyposomnia:ti,ab,kw OR sleeplessness:ti,ab,kw OR 'sleep initiation and maintenance disorders':ti,ab,kw

3. 'chronic kidney failure'/exp OR (chronic:ti,ab,kw AND (renal:ti,ab,kw OR kidney:ti,ab,kw) AND (disorder:ti,ab,kw OR disease:ti,ab,kw OR insufficiency:ti,ab,kw OR failure:ti,ab,kw)) OR (chronic:ti,ab,kw AND nephropathy:ti,ab,kw) OR (kidney:ti,ab,kw AND function,:ti,ab,kw AND chronic:ti,ab,kw AND disease:ti,ab,kw)

4. ‘kidney transplantation’/exp OR (kidney AND (allograft OR cadaver) AND transplantation):ti,ab,kw OR ((kidney OR renal) AND (allotransplantation OR grafting OR homotransplantation OR retransplantation OR homotransplantation OR transplantation)):ti,ab,kw OR (second set kidney transplantation):ti,ab,kw

5. #1 OR #2

6. #3 OR #4

7. #5 AND #6

8. #7 AND 'article'/it

*Search strategy for Cochrane Library*

1. exp sleep apnea, obstructive/ or ((sleep and (apnea or apnoea)) or OSAHS).tw.

2. exp "Sleep Initiation and Maintenance Disorders"/ or (DIMS or (Disorders of Initiating and Maintaining Sleep) or insomnia* or sleeplessness or (sleep and dysfunction*) or (early and awakening)).tw.

3. exp kidney failure, chronic/ or (((kidney or renal) and (disease or failure) and (end-stage or end stage or chronic)) or ESRD).tw.

4. exp kidney transplantation/ or (((renal or kidney) and transplant*) or (kidney and grafting)).tw.

4. 1 or 2

5. 3 or 4

6. 5 and 6

*Search strategy for CINAHL*

1. (MH "sleep apnea, obstructive+") OR (((TI sleep OR AB sleep) AND ((TI apnea OR AB apnea) OR (TI apnoea OR AB apnoea))) OR (TI OSAHS OR AB OSAHS))

2. (MH "Sleep Initiation and Maintenance Disorders+") OR ((TI DIMS OR AB DIMS) OR ((TI "Disorders of Initiating" OR AB "Disorders of Initiating") AND (TI "Maintaining Sleep" OR AB "Maintaining Sleep")) OR (TI insomnia* OR AB insomnia*) OR (TI sleeplessness OR AB sleeplessness) OR ((TI sleep OR AB sleep) AND (TI dysfunction* OR AB dysfunction*)) OR ((TI early OR AB early) AND (TI awakening OR AB awakening)))

3. (MH "kidney failure, chronic+") OR ((((TI kidney OR AB kidney) OR (TI renal OR AB renal)) AND ((TI disease OR AB disease) OR (TI failure OR AB failure)) AND ((TI end-stage OR AB end-stage) OR (TI "end stage" OR AB "end stage") OR (TI chronic OR AB chronic))) OR (TI ESRD OR AB ESRD))

4. (MH "kidney transplantation+") OR ((((TI renal OR AB renal) OR (TI kidney OR AB kidney)) AND (TI transplant* OR AB transplant*)) OR ((TI kidney OR AB kidney) AND (TI grafting OR AB grafting)))

5. 1 OR 2

6. 3 OR 4

7. 5 AND 5
