# Supplementary material for: Bidirectional association of sleep disorders with chronic kidney disease: a systematic review and meta-analysis
Source: Clin Kidney J. 2024 Oct 18;17(11):sfae279. doi: 10.1093/ckj/sfae279 (PMC11549560; doi:10.1093/ckj/sfae279)
Supplement: sfae279_Supplemental_Files [file sfae279_supplemental_files.zip › S3. Bubble Plots.pdf]

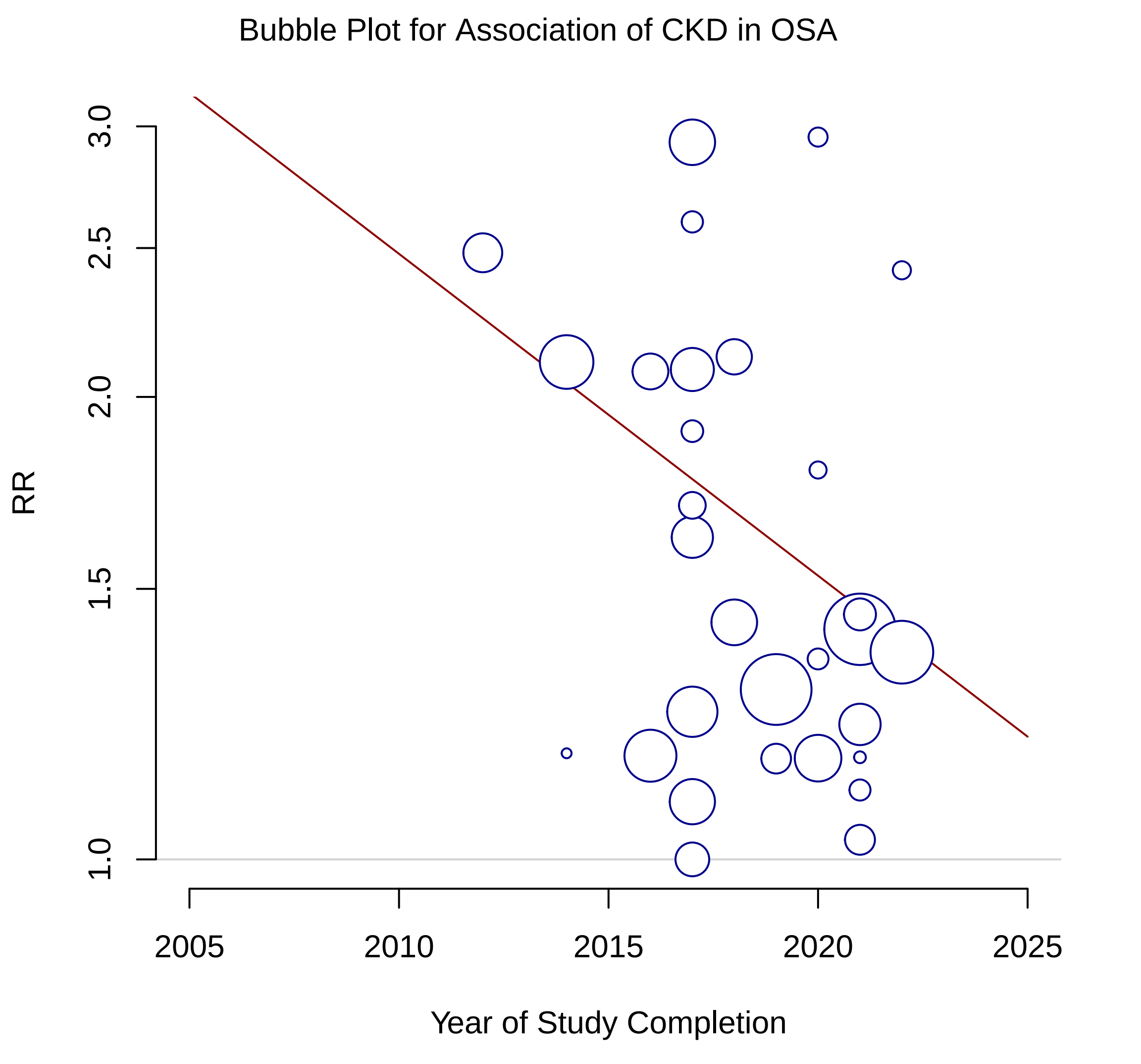
Bubble Plot for Association of CKD in OSA
RR
3.0
2.5
2.0
1.5
1.0
2005
2010
2015
2020
2025
Year of Study Completion

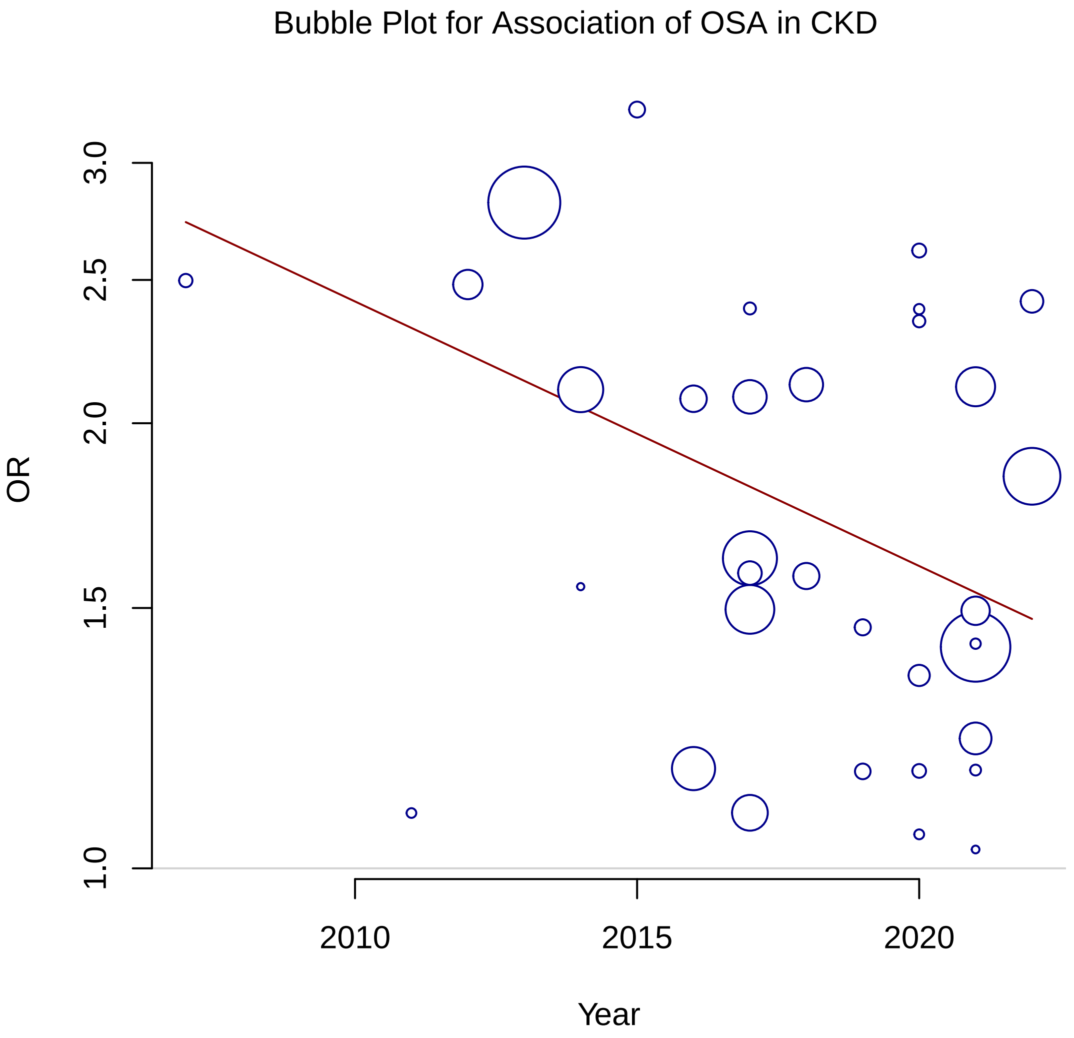

Bubble Plot for Association of OSA in CKD
OR
3.0
2.5
2.0
1.5
1.0
2010
2015
2020
Year

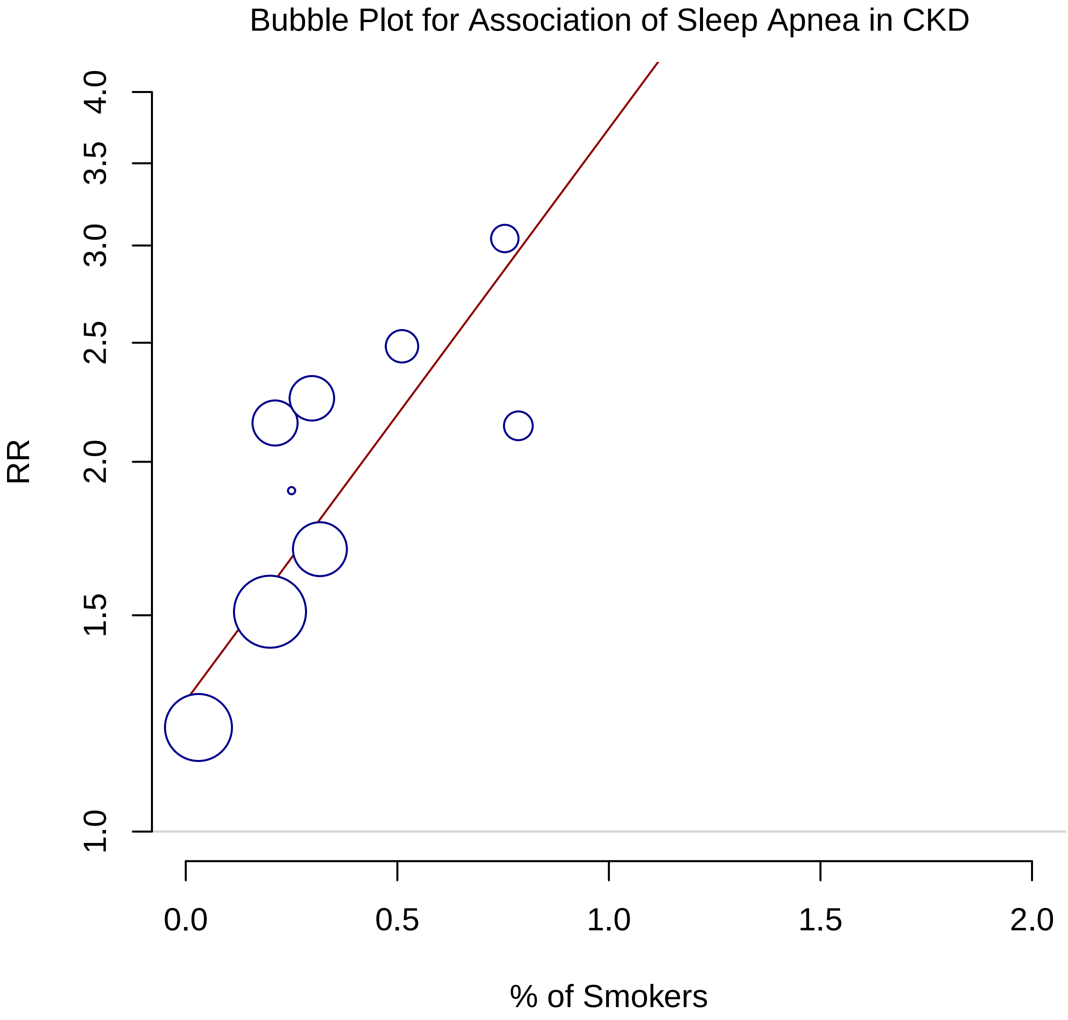

Bubble Plot for Association of Sleep Apnea in CKD
RR
1.0
1.5
2.0
2.5
3.0
3.5
4.0
0.0
0.5
1.0
1.5
2.0
% of Smokers
