# Supplementary material for: Bidirectional association of sleep disorders with chronic kidney disease: a systematic review and meta-analysis
Source: Clin Kidney J. 2024 Oct 18;17(11):sfae279. doi: 10.1093/ckj/sfae279 (PMC11549560; doi:10.1093/ckj/sfae279)
Supplement: sfae279_Supplemental_Files [file sfae279_supplemental_files.zip › S4. Results of the Meta Regression Analysis.docx]

**Supplement 4.** Results of the meta-regression analysis

*Sleep disorders as a risk factor for chronic kidney disease*

| **Outcome** | **Adjusted RR** | **SE** | **Z** | ***P*** | **95% CI Lower** | **95% CI Upper** | ***R^2^ (%)*** | ***I^2^ (%)*** |
| --- | --- | --- | --- | --- | --- | --- | --- | --- |
| Obstructive Sleep Apnea |  |  |  |  |  |  |  |  |
| Mean age (years) | 0.98 | 0.0121 | -1.8942 | 0.0900 | 0.95 | 1.00 | 9.44 | 47.46 |
| Year of study completion | 0.95 | 0.0224 | -2.1562 | 0.0380 | 0.91 | 0.96 | 23.58 | 39.87 |
| Male (%) | 0.77 | 0.5576 | -0.4798 | 0.6490 | 0.26 | 2.28 | 0.00 | 51.74 |
| Mean BMI (kg/m^2^) | 0.99 | 0.0238 | -0.2235 | 0.8820 | 0.95 | 1.04 | 0.00 | 57.66 |
| Mean AHI | 1.04 | 0.0264 | 1.6386 | 0.1667 | 0.99 | 1.10 | 54.21 | 39.51 |
| Mean eGFR | 1.01 | 0.0173 | 0.4075 | 0.7010 | 0.97 | 1.04 | 0.00 | 47.17 |
| Hypertension (%) | 0.84 | 0.4764 | -0.3562 | 0.7470 | 0.33 | 2.15 | 0.00 | 45.41 |
| Hyperlipidemia (%) | 0.94 | 0.6068 | -0.0957 | 0.9240 | 0.29 | 3.10 | 0.00 | 31.62 |
| Diabetes (%) | 0.79 | 0.4768 | -0.4940 | 0.6380 | 0.31 | 2.01 | 0.00 | 36.12 |
| CVD (%) | 0.43 | 0.8841 | -0.9421 | 0.3462 | 0.08 | 2.46 | 0.00 | 62.64 |
| Smokers (%) | 0.34 | 0.7601 | -1.4182 | 0.1930 | 0.08 | 1.51 | 7.27 | 49.42 |
| Dialysis (%) | 0.46 | 1.0627 | -0.7334 | 0.4833 | 0.06 | 3.68 | 0.00 | 53.87 |
| Hemodialysis (%) | 9.25 | 1.4723 | 1.5109 | 0.5000 | 0.52 | 165.70 | 72.36 | 31.43 |
| Peritoneal dialysis (%) | 0.00 | 7.6312 | -1.9386 | 0.3333 | 0.00 | 1.18 | 100.00 | 0.00 |
| Renal transplant (%) | 1.27 | 0.2396 | 0.9928 | 0.3510 | 0.79 | 2.03 | 0.12 | 46.96 |
| ESRD (%) | 2.49 | 0.4408 | 2.0670 | 0.0387 | 1.05 | 5.90 | 76.80 | 14.26 |
|  |  |  |  |  |  |  |  |  |
| Sleep Apnea |  |  |  |  |  |  |  |  |
| Mean age (years) | 0.99 | 0.0122 | -1.2283 | 0.2850 | 0.96 | 1.01 | 0.00 | 97.42 |
| Year of study completion | 0.99 | 0.0404 | -0.2303 | 0.7890 | 0.92 | 1.07 | 16.30 | 98.45 |
| Male (%) | 3.84 | 1.3072 | 1.0284 | 0.2800 | 0.30 | 49.71 | 98.33 | 14.87 |
| Mean BMI (kg/m^2^) | 0.98 | 0.352 | -0.6816 | 0.5330 | 0.91 | 1.05 | 0.00 | 46.37 |
| Mean AHI | 0.97 | 0.0152 | -1.8764 | 0.0847 | 0.94 | 1.00 | 63.60 | 29.74 |
| Mean eGFR | 0.99 | 0.0136 | -1.0987 | 0.3917 | 0.96 | 1.01 | 0.00 | 86.53 |
| Hypertension (%) | 0.92 | 0.5613 | -1.4307 | 0.2420 | 0.15 | 1.35 | 0.00 | 97.44 |
| Hyperlipidemia (%) | 0.80 | 0.7130 | -0.3172 | 0.8236 | 0.20 | 3.23 | 0.00 | 92.06 |
| Diabetes (%) | 0.20 | 1.1633 | -1.4003 | 0.1750 | 0.02 | 1.92 | 26.40 | 98.64 |
| CVD (%) | 0.95 | 0.4596 | -0.1204 | 0.9120 | 0.38 | 2.33 | 0.00 | 76.99 |
| Smokers (%) | 1.52 | 0.4290 | 0.9820 | 0.2790 | 0.66 | 3.53 | 0.00 | 0.00 |
| Dialysis (%) | 0.87 | 0.5585 | -0.2436 | 0.7917 | 0.29 | 2.61 | 0.00 | 60.50 |
| Hemodialysis (%) | 0.71 | 0.1697 | -2.0465 | 0.0540 | 0.51 | 0.99 | 19.86 | 51.84 |
| Peritoneal dialysis (%) | 2.05 | 0.2180 | 3.3030 | 0.5000 | 1.34 | 3.15 | 1.00 | 0.00 |
| Renal transplant (%) | 1.40 | 0.4590 | 0.7320 | 0.4642 | 0.57 | 3.44 | 0.00 | 65.77 |
| ESRD (%) | 0.15 | 2.2207 | -0.8630 | 0.3333 | 0.00 | 11.43 | 0.00 | 0.00 |

AHI, apnea hypopnea index; BMI, body-mass index; CVD, cardiovascular disease; eGFR, estimated glomerular filtration rate

*Chronic kidney disease as a risk factor for sleep disorders*

| **Outcome** | **Coefficient** | **SE** | **Z** | ***P*** | **95% CI Lower** | **95% CI Upper** | ***R^2^ (%)*** | ***I^2^ (%)*** |
| --- | --- | --- | --- | --- | --- | --- | --- | --- |
| Obstructive Sleep Apnea |  |  |  |  |  |  |  |  |
| Mean age (years) | 0.98 | 0.0076 | 1.1174 | 0.3150 | 0.95 | 1.00 | 12.43 | 26.58 |
| Year of study completion | 0.95 | 0.0149 | -2.7680 | 0.0500 | 0.91 | 0.96 | 73.23 | 11.36 |
| Male (%) | 0.77 | 0.2506 | 1.4939 | 0.2350 | 0.26 | 2.28 | 45.23 | 13.45 |
| Mean BMI (kg/m^2^) | 0.99 | 0.0166 | -0.0551 | 0.9530 | 0.95 | 1.04 | 0.00 | 54.24 |
| Mean AHI | 1.04 | 0.0147 | -0.0506 | 0.9444 | 0.99 | 1.10 | 0.00 | 26.50 |
| Mean eGFR | 1.01 | 0.0111 | -0.7252 | 0.4830 | 0.97 | 1.04 | 0.00 | 0.00 |
| Hypertension (%) | 0.84 | 0.4148 | -0.7129 | 0.5440 | 0.33 | 2.15 | 0.00 | 36.85 |
| Hyperlipidemia (%) | 0.94 | 0.3868 | 0.7628 | 0.5610 | 0.29 | 3.10 | 19.85 | 24.22 |
| Diabetes (%) | 0.79 | 0.4640 | 0.0728 | 0.9480 | 0.31 | 2.01 | 0.00 | 38.43 |
| CVD (%) | 0.43 | 0.4477 | 2.1783 | 0.1390 | 0.08 | 2.46 | 72.21 | 16.09 |
| Smokers (%) | 0.34 | 0.7546 | -1.0418 | 0.3900 | 0.08 | 1.51 | 17.20 | 38.85 |
| Dialysis (%) | 0.46 | 0.7283 | 1.0778 | 0.4500 | 0.06 | 3.68 | 0.00 | 0.00 |
| Hemodialysis (%) | 9.25 | 1.9179 | -1.2722 | 0.3333 | 0.52 | 165.70 | 0.00 | 0.00 |
| Peritoneal dialysis (%) | 0.00 | 11.7657 | 1.1000 | 0.3333 | 0.00 | 1.18 | 0.00 | 0.00 |
| Renal transplant (%) | 1.27 | 0.2706 | 0.7898 | 0.4310 | 0.79 | 2.03 | 0.00 | 34.84 |
| ESRD (%) | 1.67 | 0.7803 | 0.6586 | 0.5200 | 0.36 | 7.72 | 0.00 | 44.87 |
|  |  |  |  |  |  |  |  |  |
| Sleep Apnea |  |  |  |  |  |  |  |  |
| Mean age (years) | 0.99 | 0.0094 | -0.4881 | 0.6570 | 0.96 | 1.01 | 0.00 | 62.20 |
| Year of study completion | 0.99 | 0.0255 | 0.2799 | 0.7710 | 0.92 | 1.07 | 0.00 | 56.81 |
| Male (%) | 3.84 | 0.8461 | 1.3792 | 0.2260 | 0.30 | 49.71 | 0.00 | 59.08 |
| Mean BMI (kg/m^2^) | 0.98 | 0.0382 | -2.1488 | 0.0760 | 0.91 | 1.05 | 24.61 | 64.04 |
| Mean AHI | 0.97 | 0.0121 | 1.0436 | 0.5292 | 0.94 | 1.00 | 0.00 | 0.00 |
| Mean eGFR | 0.99 | 0.0144 | 0.9738 | 0.4500 | 0.96 | 1.01 | 0.00 | 83.61 |
| Hypertension (%) | 0.92 | 0.4968 | -1.4786 | 0.1940 | 0.15 | 1.35 | 0.00 | 64.89 |
| Hyperlipidemia (%) | 0.80 | 0.5288 | -0.4509 | 0.6200 | 0.20 | 3.23 | 0.00 | 65.11 |
| Diabetes (%) | 0.20 | 0.7874 | -0.8221 | 0.4540 | 0.02 | 1.92 | 0.00 | 64.96 |
| CVD (%) | 0.95 | 0.3327 | 0.2124 | 0.8480 | 0.38 | 2.33 | 0.00 | 63.39 |
| Smokers (%) | 1.52 | 0.3184 | 3.3694 | 0.0290 | 0.66 | 0.83 | 100.00 | 0.00 |
| Dialysis (%) | 0.87 | 0.4585 | -0.7187 | 0.5140 | 0.29 | 2.61 | 0.00 | 63.34 |
| Hemodialysis (%) | 0.71 | 1.9179 | -1.2722 | 0.3333 | 0.51 | 0.99 | 0.00 | 0.00 |
| Peritoneal dialysis (%) | 2.05 | 0.5393 | -0.5018 | 1.0000 | 1.34 | 3.15 | 1.78 | 43.78 |
| Renal transplant (%) | 1.40 | 0.1697 | -2.0456 | 0.0408 | 0.57 | 0.84 | 19.86 | 51.84 |
| ESRD (%) | 0.72 | 4.2484 | -0.0787 | 0.8333 | 0.00 | 5.98 | 0.00 | 45.61 |

AHI, apnea hypopnea index; BMI, body-mass index; CVD, cardiovascular disease; eGFR, estimated glomerular filtration rate
