# Supplementary material for: Bidirectional association of sleep disorders with chronic kidney disease: a systematic review and meta-analysis
Source: Clin Kidney J. 2024 Oct 18;17(11):sfae279. doi: 10.1093/ckj/sfae279 (PMC11549560; doi:10.1093/ckj/sfae279)
Supplement: sfae279_Supplemental_Files [file sfae279_supplemental_files.zip › S5. Additional Analyses.pdf]

Study	RR	95%−CI	P−value	Tau2	Tau	I2
Omitting Chen et al. Sleep Health. 2022.	1.72	[1.50; 1.97]	< 0.01	0.0696	0.2638	48%
Omitting Jonsson et al. Clin Kidney J. 2022	1.69	[1.48; 1.93]	< 0.01	0.0647	0.2543	48%
Omitting Brgdar et al. Cureus. 2021. (Matched cohort)	1.72	[1.50; 1.96]	< 0.01	0.0637	0.2523	47%
Omitting Brgdar et al. Cureus. 2021. (Unmatched cohort)	1.70	[1.49; 1.94]	< 0.01	0.0655	0.2560	48%
Omitting Erridge et al. Obes Surg. 2021	1.70	[1.49; 1.95]	< 0.01	0.0668	0.2585	49%
Omitting Furlan et al. Sleep Med. 2021	1.71	[1.49; 1.95]	< 0.01	0.0651	0.2551	48%
Omitting Giatti et al. Ann Am Thorac Soc. 2021	1.72	[1.50; 1.96]	< 0.01	0.0657	0.2564	48%
Omitting Jackson et al. Thorax. 2021	1.72	[1.49; 1.98]	< 0.01	0.0738	0.2717	48%
Omitting Lui et al. Int J Obstet Anesth. 2021	1.64	[1.45; 1.86]	< 0.01	0.0459	0.2142	39%
Omitting Adderley et al. Diabetes Care. 2020 (Incident)	1.70	[1.49; 1.95]	< 0.01	0.0661	0.2572	48%
Omitting Adderley et al. Diabetes Care. 2020 (Prevalent)	1.72	[1.50; 1.97]	< 0.01	0.0646	0.2541	47%
Omitting Dong et al. Endocr J 2020. (Mild OSA)	1.69	[1.48; 1.94]	< 0.01	0.0661	0.2571	49%
Omitting Dong et al. Endocr J 2020. (Moderate OSA)	1.68	[1.47; 1.92]	< 0.01	0.0626	0.2503	47%
Omitting Dong et al. Endocr J 2020. (Severe OSA)	1.66	[1.46; 1.88]	< 0.01	0.0539	0.2321	43%
Omitting Hui et al. Ann Palliat Med. 2020	1.69	[1.48; 1.93]	< 0.01	0.0648	0.2546	48%
Omitting Choi et al. Clin Exp Nephrol. 2019.	1.71	[1.50; 1.96]	< 0.01	0.0652	0.2554	48%
Omitting Choi et al. Clin Exp Nephrol. 2019.	1.73	[1.50; 1.98]	< 0.01	0.0680	0.2608	46%
Omitting Abdullah et al. Am J Cardiol. 2018	1.68	[1.47; 1.93]	< 0.01	0.0651	0.2552	48%
Omitting Huang et al. Int J Environ Res Public Health. 2018	1.71	[1.49; 1.96]	< 0.01	0.0678	0.2604	48%
Omitting Adams et al. Sleep. 2017	1.68	[1.47; 1.93]	< 0.01	0.0652	0.2554	48%
Omitting Adams et al. Sleep. 2017 (AHI = 10)	1.72	[1.50; 1.96]	< 0.01	0.0627	0.2503	47%
Omitting Adams et al. Sleep. 2017 (AHI = 30)	1.68	[1.47; 1.92]	< 0.01	0.0637	0.2524	48%
Omitting Adams et al. Sleep. 2017 (AHI 10 to 19)	1.70	[1.48; 1.94]	< 0.01	0.0666	0.2580	49%
Omitting Adams et al. Sleep. 2017 (AHI 20 to 29)	1.69	[1.48; 1.94]	< 0.01	0.0660	0.2570	48%
Omitting Dou et al. Nephron. 2017. (After match)	1.72	[1.51; 1.97]	< 0.01	0.0630	0.2510	47%
Omitting Dou et al. Nephron. 2017. (Before match)	1.65	[1.45; 1.88]	< 0.01	0.0533	0.2310	43%
Omitting Lin et al. Sleep Breath. 2017	1.72	[1.50; 1.97]	< 0.01	0.0661	0.2572	48%
Omitting Linz et al. J Hypertens. 2017	1.70	[1.48; 1.95]	< 0.01	0.0677	0.2602	49%
Omitting Yayan et al. Adv Exp Med Biol. 2017	1.67	[1.46; 1.90]	< 0.01	0.0589	0.2428	46%
Omitting Kario et al. Circ J. 2016	1.68	[1.47; 1.93]	< 0.01	0.0654	0.2558	48%
Omitting Xu et al. Sleep Med. 2016	1.72	[1.51; 1.97]	< 0.01	0.0643	0.2535	47%
Omitting Molnar et al. Thorax. 2015	1.66	[1.45; 1.89]	< 0.01	0.0558	0.2361	44%
Omitting Chen et al. J Clin Endocrinol Metab. 2014	1.68	[1.47; 1.92]	< 0.01	0.0644	0.2538	47%
Omitting Leong et al. J Clin Sleep Med. 2014	1.70	[1.49; 1.94]	< 0.01	0.0656	0.2561	48%
Omitting Abdel−Kader et al. J Hypertens. 2012	1.67	[1.46; 1.91]	< 0.01	0.0618	0.2485	46%
Omitting Beecroft et al. Eur Respir J. 2007.	1.67	[1.46; 1.90]	< 0.01	0.0590	0.2430	46%
**Random effects model**	**1.69**	**[1.48; 1.93]**	**< 0.01**	**0.0636**	**0.2522**	**47%**

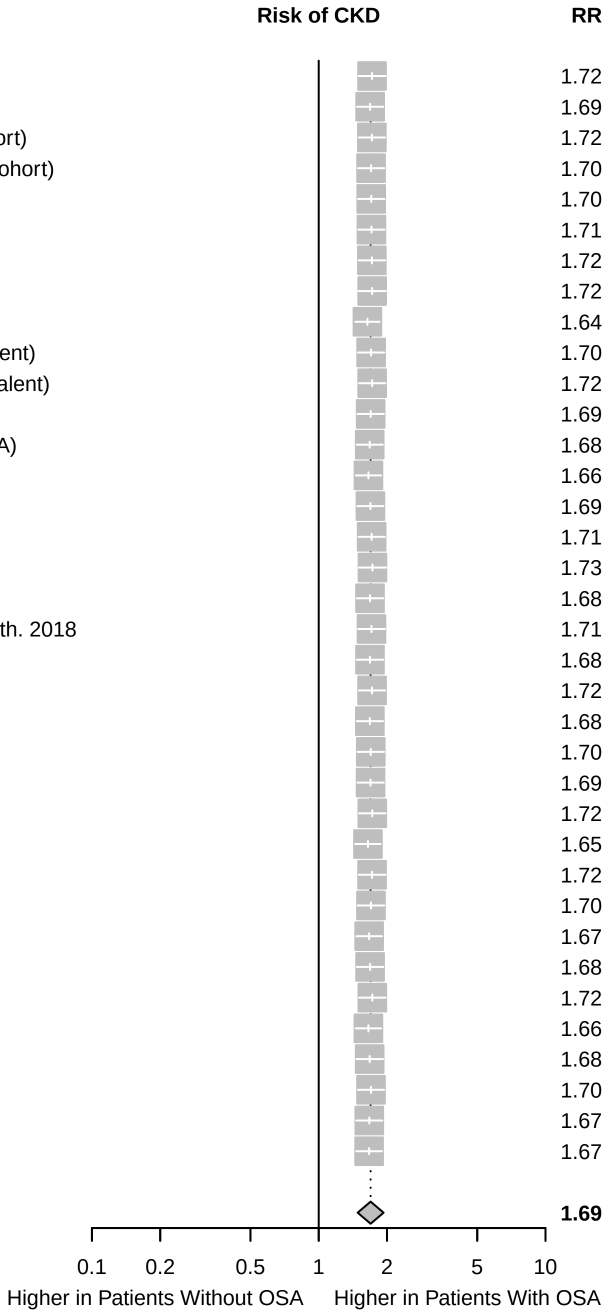

Study	RR	95%−CI	P−value	Tau2	Tau	I2
Adding Beecroft et al. Eur Respir J. 2007. (k=1)	3.73	[1.51; 9.18]	< 0.01	.	.	.
Adding Abdel−Kader et al. J Hypertens. 2012 (k=2)	2.77	[1.74; 4.42]	< 0.01	0	0	0%
Adding Chen et al. J Clin Endocrinol Metab. 2014 (k=3)	2.35	[1.75; 3.17]	< 0.01	0	0	0%
Adding Leong et al. J Clin Sleep Med. 2014 (k=4)	2.28	[1.71; 3.05]	< 0.01	0	0	0%
Adding Molnar et al. Thorax. 2015 (k=5)	2.43	[1.87; 3.17]	< 0.01	0	0	0%
Adding Kario et al. Circ J. 2016 (k=6)	2.37	[1.86; 3.01]	< 0.01	0	0	0%
Adding Xu et al. Sleep Med. 2016 (k=7)	2.08	[1.51; 2.87]	< 0.01	0.0881	0.2968	51%
Adding Adams et al. Sleep. 2017 (k=8)	2.07	[1.58; 2.71]	< 0.01	0.0612	0.2474	44%
Adding Adams et al. Sleep. 2017 (AHI = 10) (k=9)	1.93	[1.46; 2.54]	< 0.01	0.0882	0.2970	52%
Adding Adams et al. Sleep. 2017 (AHI = 30) (k=10)	1.96	[1.51; 2.55]	< 0.01	0.0790	0.2811	48%
Adding Adams et al. Sleep. 2017 (AHI 10 to 19) (k=11)	1.94	[1.52; 2.46]	< 0.01	0.0655	0.2559	42%
Adding Adams et al. Sleep. 2017 (AHI 20 to 29) (k=12)	1.93	[1.54; 2.41]	< 0.01	0.0538	0.2321	37%
Adding Dou et al. Nephron. 2017. (After match) (k=13)	1.83	[1.45; 2.29]	< 0.01	0.0736	0.2714	45%
Adding Dou et al. Nephron. 2017. (Before match) (k=14)	1.91	[1.53; 2.39]	< 0.01	0.0848	0.2913	50%
Adding Lin et al. Sleep Breath. 2017 (k=15)	1.84	[1.48; 2.28]	< 0.01	0.0868	0.2946	52%
Adding Linz et al. J Hypertens. 2017 (k=16)	1.82	[1.49; 2.22]	< 0.01	0.0754	0.2746	49%
Adding Yayan et al. Adv Exp Med Biol. 2017 (k=17)	1.87	[1.53; 2.28]	< 0.01	0.0821	0.2866	50%
Adding Abdullah et al. Am J Cardiol. 2018 (k=18)	1.88	[1.55; 2.27]	< 0.01	0.0741	0.2723	47%
Adding Huang et al. Int J Environ Res Public Health. 2018 (k=19)	1.84	[1.54; 2.20]	< 0.01	0.0680	0.2607	46%
Adding Choi et al. Clin Exp Nephrol. 2019. (k=20)	1.80	[1.51; 2.15]	< 0.01	0.0674	0.2597	45%
Adding Choi et al. Clin Exp Nephrol. 2019. (k=21)	1.75	[1.48; 2.07]	< 0.01	0.0684	0.2616	50%
Adding Adderley et al. Diabetes Care. 2020 (Incident) (k=22)	1.74	[1.47; 2.05]	< 0.01	0.0644	0.2538	47%
Adding Adderley et al. Diabetes Care. 2020 (Prevalent) (k=23)	1.70	[1.45; 1.99]	< 0.01	0.0637	0.2524	48%
Adding Dong et al. Endocr J 2020. (Mild OSA) (k=24)	1.70	[1.45; 1.98]	< 0.01	0.0599	0.2447	45%
Adding Dong et al. Endocr J 2020. (Moderate OSA) (k=25)	1.72	[1.47; 2.00]	< 0.01	0.0610	0.2469	45%
Adding Dong et al. Endocr J 2020. (Severe OSA) (k=26)	1.77	[1.51; 2.07]	< 0.01	0.0741	0.2722	49%
Adding Hui et al. Ann Palliat Med. 2020 (k=27)	1.77	[1.52; 2.08]	< 0.01	0.0721	0.2686	48%
Adding Brgdar et al. Cureus. 2021. (Matched cohort) (k=28)	1.74	[1.49; 2.04]	< 0.01	0.0726	0.2694	48%
Adding Brgdar et al. Cureus. 2021. (Unmatched cohort) (k=29)	1.73	[1.49; 2.02]	< 0.01	0.0699	0.2644	46%
Adding Erridge et al. Obes Surg. 2021 (k=30)	1.72	[1.48; 1.99]	< 0.01	0.0655	0.2559	44%
Adding Furlan et al. Sleep Med. 2021 (k=31)	1.70	[1.47; 1.97]	< 0.01	0.0637	0.2524	43%
Adding Giatti et al. Ann Am Thorac Soc. 2021 (k=32)	1.68	[1.46; 1.93]	< 0.01	0.0613	0.2475	42%
Adding Jackson et al. Thorax. 2021 (k=33)	1.65	[1.45; 1.89]	< 0.01	0.0517	0.2274	41%
Adding Lui et al. Int J Obstet Anesth. 2021 (k=34)	1.71	[1.49; 1.97]	< 0.01	0.0709	0.2662	49%
Adding Chen et al. Sleep health. 2022. (k=35)	1.69	[1.48; 1.93]	< 0.01	0.0647	0.2543	48%
Adding Jonsson et al. Clin Kidney J. 2022 (k=36)	1.69	[1.48; 1.93]	< 0.01	0.0636	0.2522	47%
**Random effects model**	**1.69**	**[1.48; 1.93]**	**< 0.01**	**0.0636**	**0.2522**	**47%**

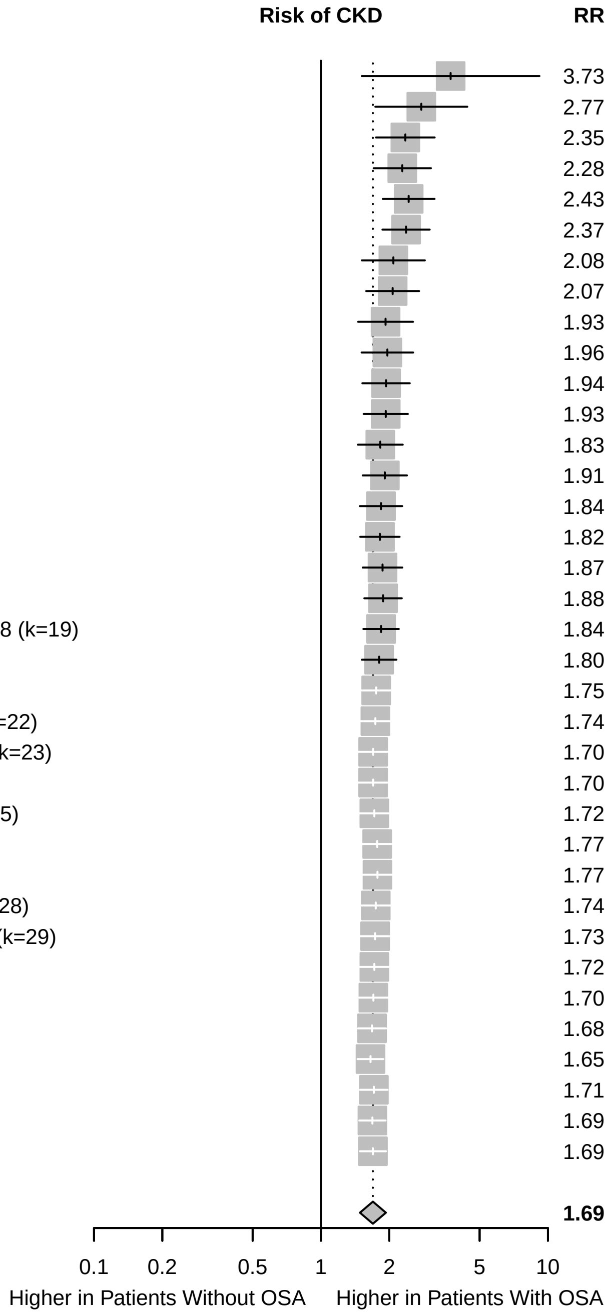

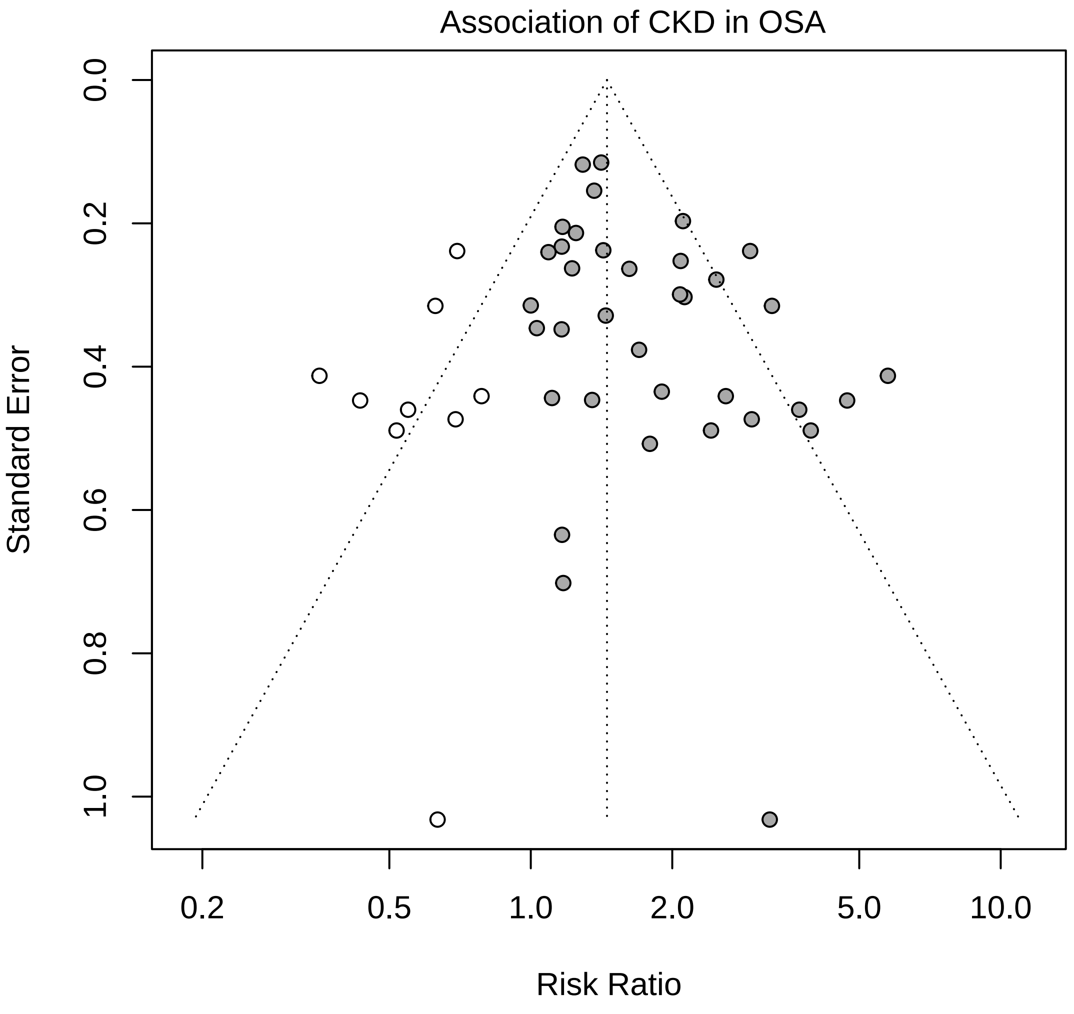

Association of CKD in OSA
Standard Error
0.0
0.2
0.4
0.6
0.8
1.0
0.2
0.5
1.0
2.0
5.0
10.0
Risk Ratio

Study	RR	95%−CI	P−value	Tau2	Tau	I2
Omitting Ding et al. Int. J. Environ. Res. Public Health. 2021.	1.64	[1.37; 1.96]	< 0.01	0.0894	0.2990	66%
Omitting Wang et al. BMJ Open. 2021	1.70	[1.42; 2.04]	< 0.01	0.0878	0.2963	63%
Omitting Koo et al. Heart. 2020	1.66	[1.38; 1.99]	< 0.01	0.0940	0.3067	66%
Omitting Mehta et al. Bone. 2018	1.70	[1.42; 2.03]	< 0.01	0.0848	0.2911	64%
Omitting Yatsu et al. Clin Res Cardiol. 2018	1.63	[1.36; 1.96]	< 0.01	0.0904	0.3007	65%
Omitting Yeo et al. Ann Transl Med. 2017	1.68	[1.41; 2.02]	< 0.01	0.0903	0.3005	66%
Omitting Bhandari et al. Respirology. 2016.	1.70	[1.42; 2.04]	< 0.01	0.0862	0.2936	64%
Omitting Chang et al. Medicine. 2016.	1.64	[1.37; 1.96]	< 0.01	0.0904	0.3007	66%
Omitting Chang et al. Medicine. 2016.	1.66	[1.38; 1.99]	< 0.01	0.0930	0.3049	66%
Omitting Chu et al. Respirology. 2016	1.60	[1.35; 1.90]	< 0.01	0.0724	0.2691	59%
Omitting Elias et al. Sleep Med. 2016	1.67	[1.40; 2.00]	< 0.01	0.0911	0.3019	66%
Omitting Xu et al. Sleep Med. 2016	1.71	[1.43; 2.04]	< 0.01	0.0843	0.2904	63%
Omitting Lee et al. Sleep. 2015	1.62	[1.35; 1.94]	< 0.01	0.0828	0.2878	59%
Omitting Furukawa et al. Eur J Endocrinol. 2013	1.63	[1.36; 1.96]	< 0.01	0.0921	0.3035	65%
Omitting Chou et al. Am J Ophthalmol. 2012	1.73	[1.46; 2.04]	< 0.01	0.0646	0.2542	55%
Omitting Nicholl et al. Chest. 2012	1.64	[1.37; 1.96]	< 0.01	0.0903	0.3005	66%
Omitting Platinga et al. Am J Kidney Dis. 2011	1.67	[1.39; 2.01]	< 0.01	0.0959	0.3097	66%
Omitting Roumelioti et al. Clin J Am Soc Nephrol. 2011	1.63	[1.36; 1.95]	< 0.01	0.0881	0.2968	65%
Omitting Jurado−Gamez et al. Nephron Clin Pract. 2007	1.65	[1.37; 1.98]	< 0.01	0.0932	0.3052	66%
**Random effects model**	**1.66**	**[1.39; 1.98]**	**< 0.01**	**0.0878**	**0.2963**	**64%**

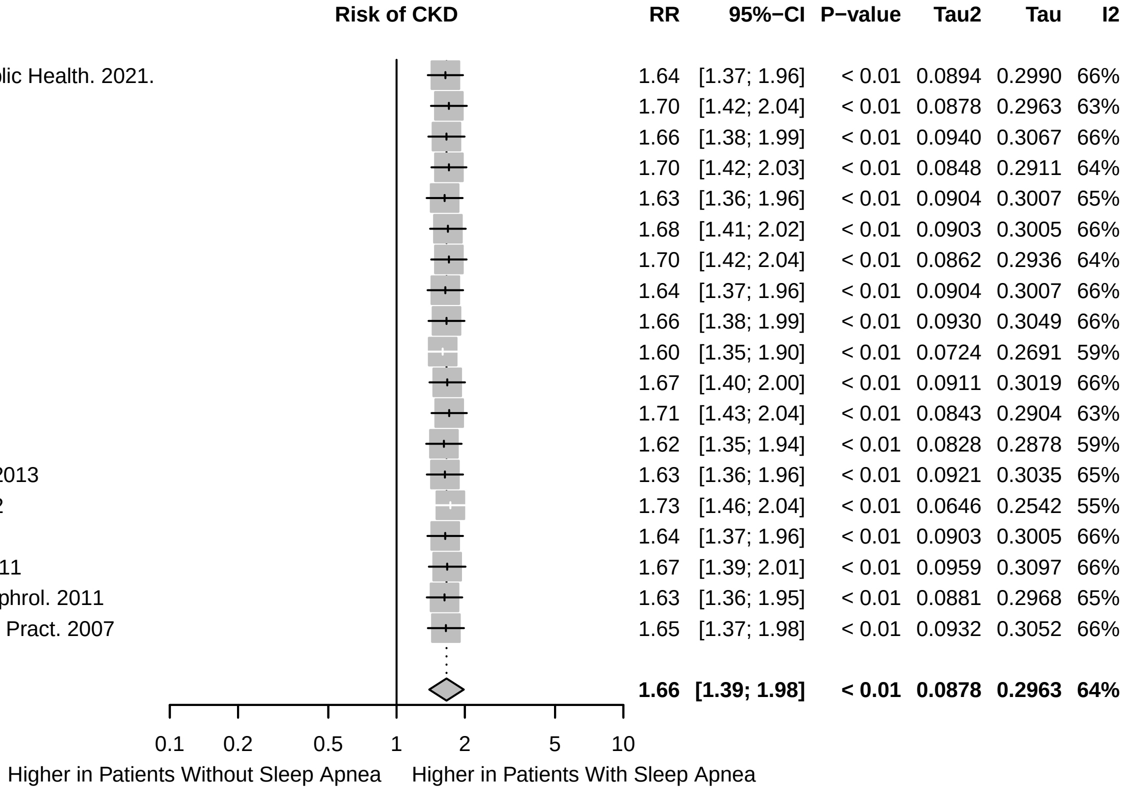

Study	RR	95%−CI	P−value	Tau2	Tau	I2
Adding Jurado−Gamez et al. Nephron Clin Pract. 2007 (k=1)	1.89	[1.08; 3.33]	0.03	.	.	.
Adding Platinga et al. Am J Kidney Dis. 2011 (k=2)	1.63	[1.17; 2.28]	< 0.01	0	0	0%
Adding Roumelioti et al. Clin J Am Soc Nephrol. 2011 (k=3)	1.83	[1.37; 2.44]	< 0.01	0.0012	0.0350	2%
Adding Chou et al. Am J Ophthalmol. 2012 (k=4)	1.56	[1.05; 2.33]	0.03	0.1113	0.3336	70%
Adding Nicholl et al. Chest. 2012 (k=5)	1.66	[1.15; 2.39]	< 0.01	0.1104	0.3323	67%
Adding Furukawa et al. Eur J Endocrinol. 2013 (k=6)	1.74	[1.26; 2.40]	< 0.01	0.1043	0.3229	67%
Adding Lee et al. Sleep. 2015 (k=7)	1.82	[1.37; 2.43]	< 0.01	0.1010	0.3178	73%
Adding Bhandari et al. Respirology. 2016. (k=8)	1.71	[1.30; 2.25]	< 0.01	0.1051	0.3242	73%
Adding Chang et al. Medicine. 2016. (k=9)	1.75	[1.35; 2.26]	< 0.01	0.0979	0.3130	70%
Adding Chang et al. Medicine. 2016. (k=10)	1.74	[1.37; 2.21]	< 0.01	0.0877	0.2962	66%
Adding Chu et al. Respirology. 2016 (k=11)	1.84	[1.45; 2.32]	< 0.01	0.0981	0.3132	69%
Adding Elias et al. Sleep Med. 2016 (k=12)	1.81	[1.44; 2.26]	< 0.01	0.0937	0.3062	67%
Adding Xu et al. Sleep Med. 2016 (k=13)	1.73	[1.39; 2.15]	< 0.01	0.1018	0.3190	69%
Adding Yeo et al. Ann Transl Med. 2017 (k=14)	1.70	[1.37; 2.09]	< 0.01	0.0986	0.3140	68%
Adding Mehta et al. Bone. 2018 (k=15)	1.64	[1.34; 2.02]	< 0.01	0.1024	0.3200	68%
Adding Yatsu et al. Clin Res Cardiol. 2018 (k=16)	1.68	[1.38; 2.04]	< 0.01	0.0972	0.3118	67%
Adding Koo et al. Heart. 2020 (k=17)	1.68	[1.40; 2.02]	< 0.01	0.0899	0.2999	65%
Adding Ding et al. Int. J. Environ. Res. Public Health. 2021. (k=18)	1.70	[1.42; 2.04]	< 0.01	0.0878	0.2963	63%
Adding Wang et al. BMJ Open. 2021 (k=19)	1.66	[1.39; 1.98]	< 0.01	0.0878	0.2963	64%
**Random effects model**	**1.66**	**[1.39; 1.98]**	**< 0.01**	**0.0878**	**0.2963**	**64%**

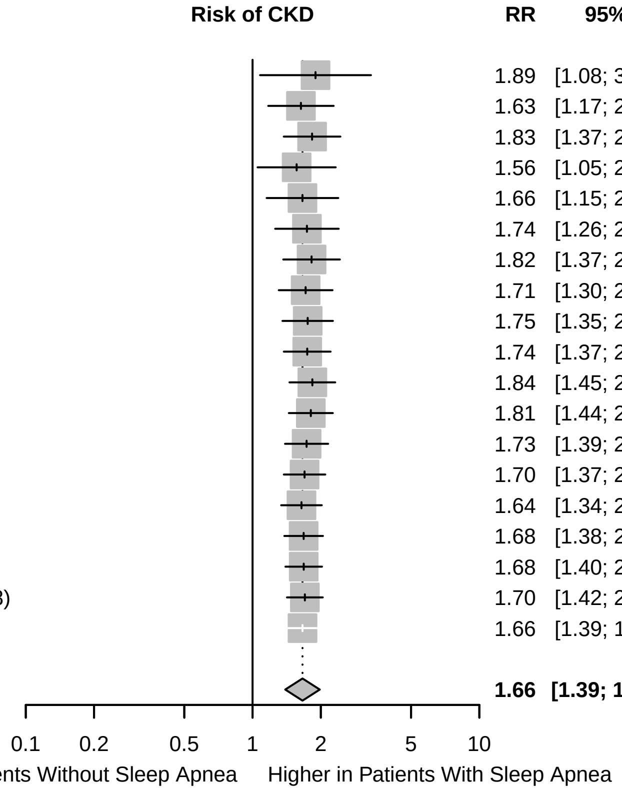

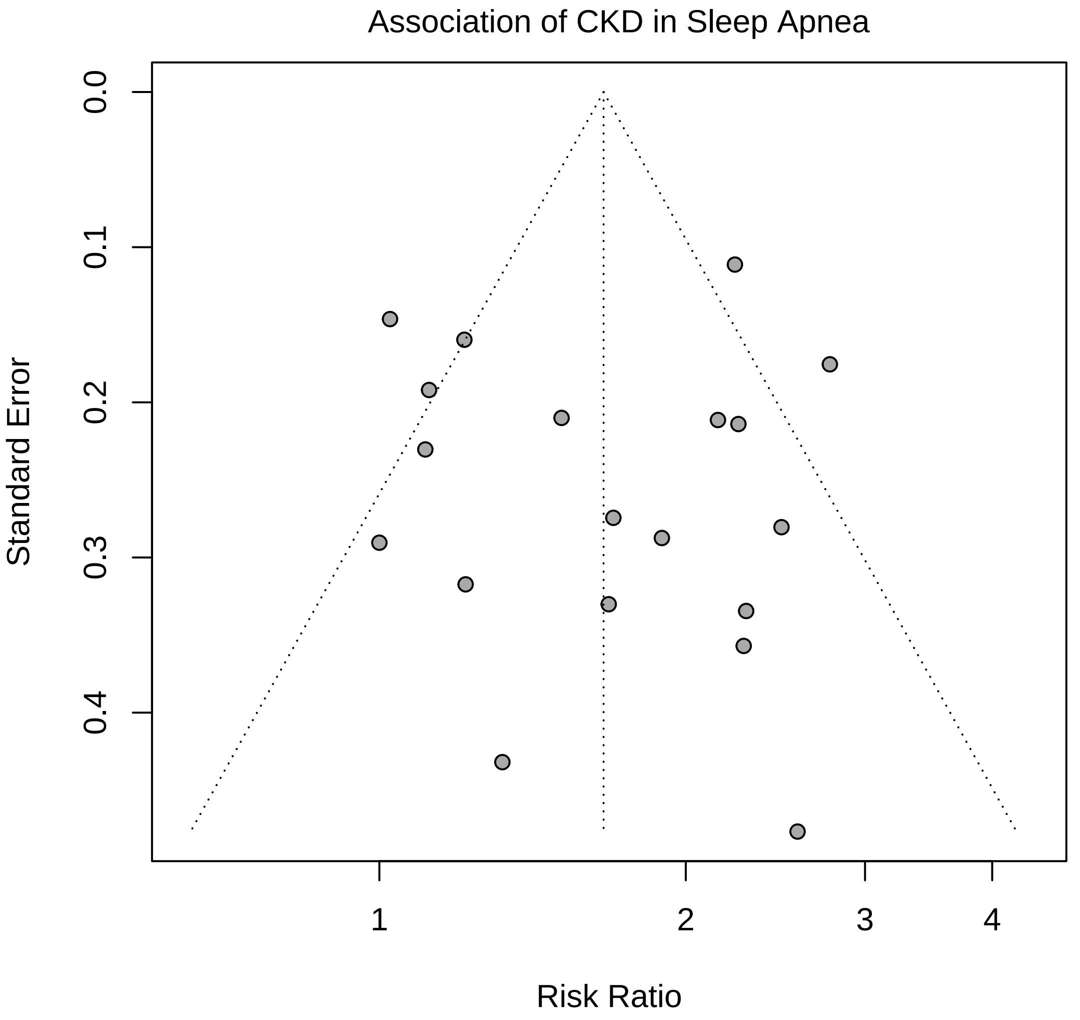

Association of CKD in Sleep Apnea
0.0
0.1
0.2
0.3
0.4
Standard Error
1
2
3
4
Risk Ratio

Study	RR	95%−CI	P−value	Tau2	Tau	I2
Omitting Chen et al. Sleep Health. 2022.	1.73	[1.53; 1.96]	< 0.01	0.0389	0.1971	36%
Omitting Jonsson et al. Clin Kidney J. 2022	1.73	[1.53; 1.94]	< 0.01	0.0347	0.1863	35%
Omitting Brgdar et al. Cureus. 2021. (Matched cohort)	1.75	[1.55; 1.97]	< 0.01	0.0343	0.1852	35%
Omitting Brgdar et al. Cureus. 2021. (Unmatched cohort)	1.75	[1.55; 1.97]	< 0.01	0.0346	0.1860	35%
Omitting Erridge et al. Obes Surg. 2021	1.74	[1.55; 1.96]	< 0.01	0.0356	0.1886	36%
Omitting Furlan et al. Sleep Med. 2021	1.75	[1.55; 1.97]	< 0.01	0.0358	0.1892	35%
Omitting Giatti et al. Ann Am Thorac Soc. 2021	1.76	[1.57; 1.98]	< 0.01	0.0324	0.1800	33%
Omitting Jackson et al. Thorax. 2021	1.77	[1.57; 2.00]	< 0.01	0.0300	0.1732	29%
Omitting Lui et al. Int J Obstet Anesth. 2021	1.72	[1.53; 1.95]	< 0.01	0.0360	0.1898	35%
Omitting Adderley et al. Diabetes Care. 2020 (Incident)	1.75	[1.55; 1.97]	< 0.01	0.0350	0.1870	35%
Omitting Adderley et al. Diabetes Care. 2020 (Prevalent)	1.75	[1.56; 1.97]	< 0.01	0.0342	0.1850	35%
Omitting Dong et al. Endocr J 2020. (Mild OSA)	1.75	[1.56; 1.97]	< 0.01	0.0341	0.1846	35%
Omitting Dong et al. Endocr J 2020. (Moderate OSA)	1.73	[1.54; 1.95]	< 0.01	0.0354	0.1881	35%
Omitting Dong et al. Endocr J 2020. (Severe OSA)	1.73	[1.54; 1.95]	< 0.01	0.0346	0.1861	35%
Omitting Hui et al. Ann Palliat Med. 2020	1.73	[1.54; 1.95]	< 0.01	0.0354	0.1881	35%
Omitting Choi et al. Clin Exp Nephrol. 2019.	1.75	[1.56; 1.97]	< 0.01	0.0340	0.1843	34%
Omitting Choi et al. Clin Exp Nephrol. 2019.	1.75	[1.55; 1.97]	< 0.01	0.0356	0.1888	36%
Omitting Abdullah et al. Am J Cardiol. 2018.	1.73	[1.53; 1.95]	< 0.01	0.0359	0.1896	35%
Omitting Huang et al. Int J Environ Res Public Health. 2018	1.74	[1.55; 1.97]	< 0.01	0.0362	0.1902	36%
Omitting Adams et al. Sleep. 2017.	1.73	[1.53; 1.95]	< 0.01	0.0361	0.1901	35%
Omitting Dou et al. Nephron. 2017. (Matched cohort)	1.78	[1.58; 1.99]	< 0.01	0.0279	0.1671	30%
Omitting Dou et al. Nephron. 2017. (Unmatched cohort)	1.75	[1.55; 1.98]	< 0.01	0.0358	0.1893	35%
Omitting Lin et al. Sleep Breath. 2017	1.74	[1.55; 1.97]	< 0.01	0.0361	0.1901	36%
Omitting Linz et al. J Hypertens. 2017	1.75	[1.54; 1.98]	< 0.01	0.0377	0.1942	35%
Omitting Yayan et al. Adv Exp Med Biol. 2017	1.73	[1.54; 1.95]	< 0.01	0.0353	0.1879	35%
Omitting Kario et al. Circ J. 2016	1.73	[1.53; 1.95]	< 0.01	0.0360	0.1898	36%
Omitting Xu et al. Sleep Med. 2016	1.78	[1.58; 2.00]	< 0.01	0.0280	0.1673	29%
Omitting Molnar et al. Thorax. 2015	1.72	[1.53; 1.94]	< 0.01	0.0319	0.1786	33%
Omitting Chen et al. J Clin Endocrinol Metab. 2014	1.72	[1.52; 1.94]	< 0.01	0.0362	0.1902	35%
Omitting Leong et al. J Clin Sleep Med. 2014	1.74	[1.55; 1.96]	< 0.01	0.0358	0.1891	36%
Omitting Low et al. Sleep Med. 2013	1.64	[1.50; 1.80]	< 0.01	0	0	0%
Omitting Abdel−Kader et al. J Hypertens. 2012.	1.72	[1.53; 1.94]	< 0.01	0.0338	0.1838	34%
Omitting Chou et al. Nephrol Dial Transplant. 2011	1.75	[1.55; 1.97]	< 0.01	0.0343	0.1852	35%
Omitting Beecroft et al. Eur Respir J. 2007.	1.73	[1.54; 1.95]	< 0.01	0.0350	0.1871	35%
**Random effects model**	**1.74**	**[1.55; 1.96]**	**< 0.01**	**0.0337**	**0.1836**	**34%**

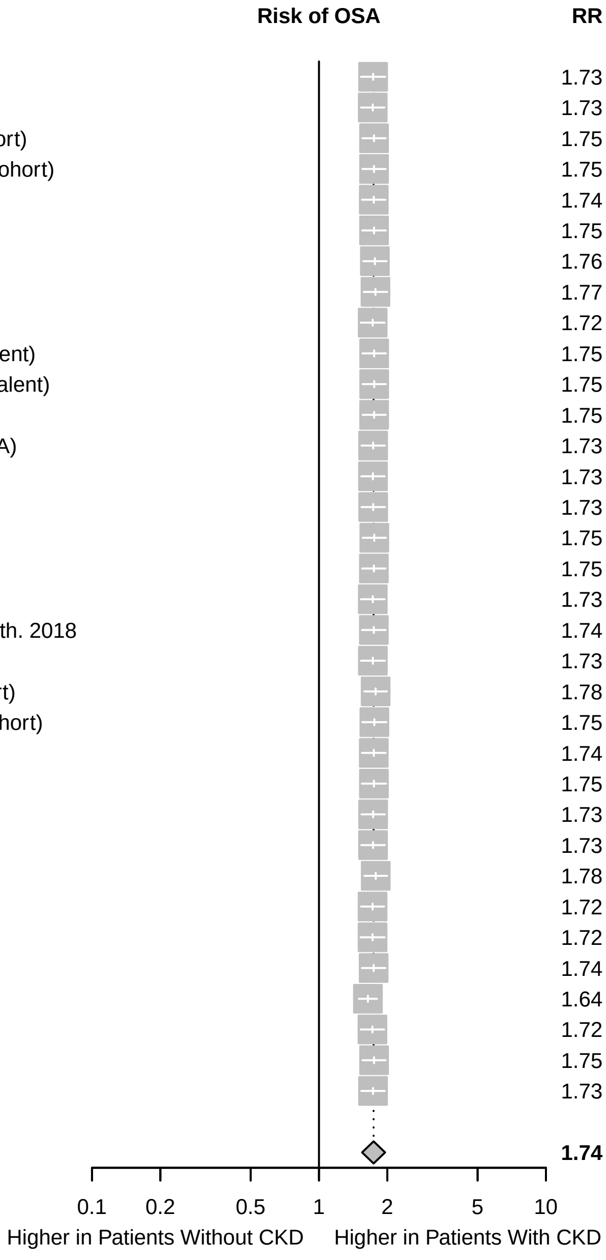

Study	RR	95%−CI	P−value	Tau2	Tau	I2
Adding Beecroft et al. Eur Respir J. 2007. (k=1)	2.50	[1.01; 6.15]	0.05	.	.	.
Adding Chou et al. Nephrol Dial Transplant. 2011 (k=2)	1.74	[0.78; 3.90]	0.18	0.0860	0.2933	25%
Adding Abdel−Kader et al. J Hypertens. 2012. (k=3)	2.18	[1.42; 3.35]	< 0.01	0	0	0%
Adding Low et al. Sleep Med. 2013 (k=4)	2.68	[2.22; 3.24]	< 0.01	0	0	0%
Adding Chen et al. J Clin Endocrinol Metab. 2014 (k=5)	2.53	[2.11; 3.04]	< 0.01	0.0028	0.0526	5%
Adding Leong et al. J Clin Sleep Med. 2014 (k=6)	2.54	[2.15; 3.01]	< 0.01	0	0	0%
Adding Molnar et al. Thorax. 2015 (k=7)	2.57	[2.18; 3.03]	< 0.01	0	0	0%
Adding Kario et al. Circ J. 2016 (k=8)	2.53	[2.16; 2.96]	< 0.01	0	0	0%
Adding Xu et al. Sleep Med. 2016 (k=9)	2.08	[1.59; 2.73]	< 0.01	0.0785	0.2802	55%
Adding Adams et al. Sleep. 2017. (k=10)	2.09	[1.64; 2.65]	< 0.01	0.0621	0.2492	50%
Adding Dou et al. Nephron. 2017. (Matched cohort) (k=11)	1.93	[1.49; 2.50]	< 0.01	0.1002	0.3165	62%
Adding Dou et al. Nephron. 2017. (Unmatched cohort) (k=12)	1.88	[1.48; 2.39]	< 0.01	0.0952	0.3085	63%
Adding Lin et al. Sleep Breath. 2017 (k=13)	1.86	[1.48; 2.33]	< 0.01	0.0881	0.2968	60%
Adding Linz et al. J Hypertens. 2017 (k=14)	1.83	[1.49; 2.25]	< 0.01	0.0766	0.2767	59%
Adding Yayan et al. Adv Exp Med Biol. 2017 (k=15)	1.85	[1.52; 2.25]	< 0.01	0.0717	0.2678	56%
Adding Abdullah et al. Am J Cardiol. 2018. (k=16)	1.87	[1.55; 2.24]	< 0.01	0.0637	0.2524	53%
Adding Huang et al. Int J Environ Res Public Health. 2018 (k=17)	1.85	[1.55; 2.21]	< 0.01	0.0593	0.2435	51%
Adding Choi et al. Clin Exp Nephrol. 2019. (k=18)	1.82	[1.53; 2.17]	< 0.01	0.0598	0.2445	50%
Adding Choi et al. Clin Exp Nephrol. 2019. (k=19)	1.81	[1.53; 2.14]	< 0.01	0.0566	0.2380	48%
Adding Adderley et al. Diabetes Care. 2020 (Incident) (k=20)	1.79	[1.52; 2.11]	< 0.01	0.0550	0.2346	46%
Adding Adderley et al. Diabetes Care. 2020 (Prevalent) (k=21)	1.77	[1.51; 2.08]	< 0.01	0.0547	0.2338	45%
Adding Dong et al. Endocr J 2020. (Mild OSA) (k=22)	1.75	[1.49; 2.06]	< 0.01	0.0545	0.2334	44%
Adding Dong et al. Endocr J 2020. (Moderate OSA) (k=23)	1.77	[1.51; 2.06]	< 0.01	0.0511	0.2261	42%
Adding Dong et al. Endocr J 2020. (Severe OSA) (k=24)	1.78	[1.53; 2.08]	< 0.01	0.0487	0.2207	40%
Adding Hui et al. Ann Palliat Med. 2020 (k=25)	1.79	[1.55; 2.08]	< 0.01	0.0456	0.2136	38%
Adding Brgdar et al. Cureus. 2021. (Matched cohort) (k=26)	1.78	[1.54; 2.06]	< 0.01	0.0451	0.2124	37%
Adding Brgdar et al. Cureus. 2021. (Unmatched cohort) (k=27)	1.77	[1.53; 2.05]	< 0.01	0.0442	0.2102	36%
Adding Erridge et al. Obes Surg. 2021 (k=28)	1.76	[1.53; 2.03]	< 0.01	0.0415	0.2038	34%
Adding Furlan et al. Sleep Med. 2021 (k=29)	1.75	[1.53; 2.01]	< 0.01	0.0389	0.1973	32%
Adding Giatti et al. Ann Am Thorac Soc. 2021 (k=30)	1.73	[1.51; 1.98]	< 0.01	0.0414	0.2035	34%
Adding Jackson et al. Thorax. 2021 (k=31)	1.70	[1.49; 1.94]	< 0.01	0.0428	0.2069	38%
Adding Lui et al. Int J Obstet Anesth. 2021 (k=32)	1.72	[1.51; 1.95]	< 0.01	0.0400	0.2001	37%
Adding Chen et al. Sleep Health. 2022. (k=33)	1.73	[1.53; 1.94]	< 0.01	0.0347	0.1863	35%
Adding Jonsson et al. Clin Kidney J. 2022 (k=34)	1.74	[1.55; 1.96]	< 0.01	0.0337	0.1836	34%
**Random effects model**	**1.74**	**[1.55; 1.96]**	**< 0.01**	**0.0337**	**0.1836**	**34%**

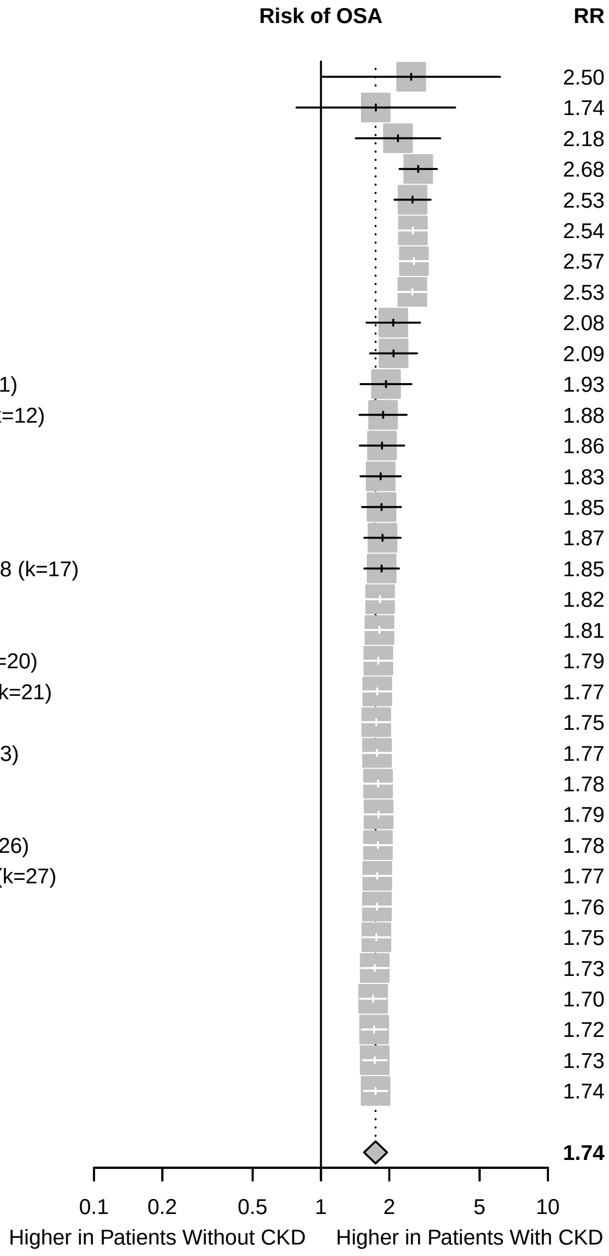

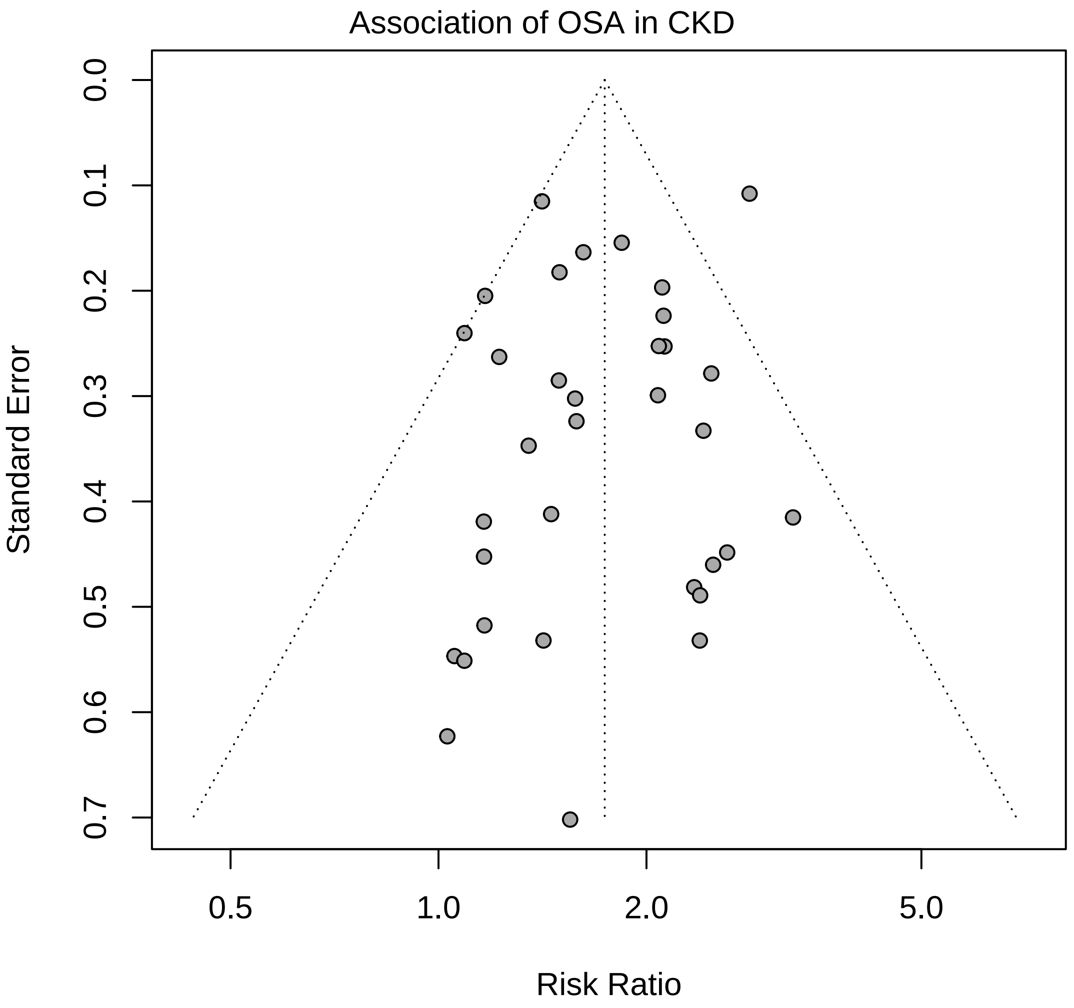
Association of OSA in CKD
Standard Error
0.0
0.1
0.2
0.3
0.4
0.5
0.6
0.7
0.5
1.0
2.0
5.0
Risk Ratio

Study	RR	95%−CI	P−value	Tau2	Tau	I2
Omitting Ding et al. Int. J. Environ. Res. Public Health. 2021.	1.48	[1.27; 1.71]	< 0.01	0.0729	0.2699	60%
Omitting Wang et al. BMJ Open. 2021	1.49	[1.28; 1.72]	< 0.01	0.0728	0.2699	60%
Omitting Koo et al. Heart. 2020	1.47	[1.26; 1.71]	< 0.01	0.0750	0.2739	60%
Omitting Mehta et al. Bone. 2018	1.51	[1.30; 1.75]	< 0.01	0.0699	0.2643	58%
Omitting Yatsu et al. Clin Res Cardiol. 2018	1.45	[1.25; 1.67]	< 0.01	0.0656	0.2561	57%
Omitting Yeo et al. Ann Transl Med. 2017	1.50	[1.29; 1.75]	< 0.01	0.0784	0.2800	59%
Omitting Bhandari et al. Respirology. 2016.	1.50	[1.29; 1.74]	< 0.01	0.0714	0.2672	60%
Omitting Chang et al. Medicine. 2016.	1.44	[1.25; 1.66]	< 0.01	0.0620	0.2490	56%
Omitting Chang et al. Medicine. 2016.	1.46	[1.26; 1.69]	< 0.01	0.0703	0.2651	59%
Omitting Chu et al. Respirology. 2016	1.46	[1.26; 1.69]	< 0.01	0.0703	0.2651	59%
Omitting Elias et al. Sleep Med. 2016	1.50	[1.30; 1.74]	< 0.01	0.0690	0.2627	59%
Omitting Xu et al. Sleep Med. 2016	1.48	[1.28; 1.72]	< 0.01	0.0743	0.2725	60%
Omitting Lee et al. Sleep. 2015	1.46	[1.26; 1.69]	< 0.01	0.0695	0.2637	59%
Omitting Furukawa et al. Eur J Endocrinol. 2013	1.45	[1.25; 1.68]	< 0.01	0.0674	0.2596	58%
Omitting Chou et al. Am J Ophthalmol. 2012	1.50	[1.29; 1.73]	< 0.01	0.0719	0.2682	60%
Omitting Nicholl et al. Chest. 2012	1.53	[1.34; 1.75]	< 0.01	0.0453	0.2127	48%
Omitting Nicholl et al. Chest. 2012 (ESRD)	1.48	[1.28; 1.72]	< 0.01	0.0744	0.2727	60%
Omitting Platinga et al. Am J Kidney Dis. 2011	1.48	[1.26; 1.73]	< 0.01	0.0841	0.2900	60%
Omitting Roumelioti et al. Clin J Am Soc Nephrol. 2011	1.45	[1.25; 1.68]	< 0.01	0.0660	0.2569	58%
Omitting Canales et al. Sleep Med. 2008.	1.48	[1.28; 1.72]	< 0.01	0.0736	0.2713	60%
Omitting Canales et al. Sleep Med. 2008.	1.49	[1.28; 1.73]	< 0.01	0.0744	0.2727	60%
Omitting Canales et al. Sleep Med. 2008.	1.46	[1.26; 1.69]	< 0.01	0.0706	0.2657	59%
Omitting Canales et al. Nephrol Dial Transplant. 2008. (Mayo Clinic)	1.49	[1.28; 1.74]	< 0.01	0.0811	0.2848	60%
Omitting Canales et al. Nephrol Dial Transplant. 2008. (MDRD)	1.52	[1.31; 1.76]	< 0.01	0.0635	0.2520	54%
Omitting Canales et al. Nephrol Dial Transplant. 2008. (Cockroft)	1.46	[1.26; 1.69]	< 0.01	0.0697	0.2640	59%
Omitting Jurado−Gamez et al. Nephron Clin Pract. 2007	1.48	[1.28; 1.71]	< 0.01	0.0724	0.2690	60%
**Random effects model**	**1.48**	**[1.28; 1.71]**	**< 0.01**	**0.0704**	**0.2654**	**59%**

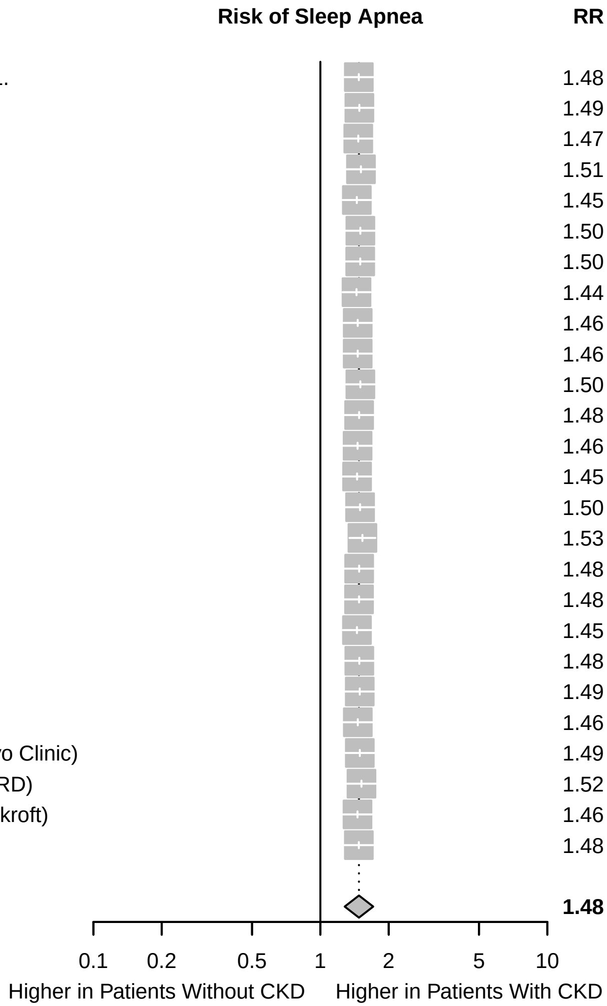

Study	RR	95%−CI	P−value	Tau2	Tau	I2
Adding Jurado−Gamez et al. Nephron Clin Pract. 2007 (k=1)	1.89	[0.33; 10.79]	0.47	.	.	.
Adding Canales et al. Sleep Med. 2008. (k=2)	1.39	[0.74; 2.63]	0.31	0	0	0%
Adding Canales et al. Sleep Med. 2008. (k=3)	1.30	[0.88; 1.92]	0.18	0	0	0%
Adding Canales et al. Sleep Med. 2008. (k=4)	1.49	[1.08; 2.07]	0.02	0	0	0%
Adding Canales et al. Nephrol Dial Transplant. 2008. (Mayo Clinic) (k=5)	1.39	[1.14; 1.70]	< 0.01	0	0	0%
Adding Canales et al. Nephrol Dial Transplant. 2008. (MDRD) (k=6)	1.25	[1.03; 1.51]	0.02	0.0108	0.1041	19%
Adding Canales et al. Nephrol Dial Transplant. 2008. (Cockroft) (k=7)	1.37	[1.09; 1.72]	< 0.01	0.0337	0.1835	42%
Adding Platinga et al. Am J Kidney Dis. 2011 (k=8)	1.39	[1.16; 1.67]	< 0.01	0.0244	0.1563	41%
Adding Roumelioti et al. Clin J Am Soc Nephrol. 2011 (k=9)	1.48	[1.21; 1.80]	< 0.01	0.0392	0.1979	51%
Adding Chou et al. Am J Ophthalmol. 2012 (k=10)	1.44	[1.19; 1.74]	< 0.01	0.0363	0.1905	48%
Adding Nicholl et al. Chest. 2012 (k=11)	1.34	[1.07; 1.68]	0.01	0.0811	0.2848	66%
Adding Nicholl et al. Chest. 2012 (ESRD) (k=12)	1.35	[1.09; 1.66]	< 0.01	0.0730	0.2703	63%
Adding Furukawa et al. Eur J Endocrinol. 2013 (k=13)	1.41	[1.14; 1.73]	< 0.01	0.0812	0.2850	66%
Adding Lee et al. Sleep. 2015 (k=14)	1.45	[1.18; 1.77]	< 0.01	0.0831	0.2883	65%
Adding Bhandari et al. Respirology. 2016. (k=15)	1.42	[1.17; 1.72]	< 0.01	0.0802	0.2833	63%
Adding Chang et al. Medicine. 2016. (k=16)	1.48	[1.21; 1.80]	< 0.01	0.0939	0.3064	66%
Adding Chang et al. Medicine. 2016. (k=17)	1.51	[1.24; 1.83]	< 0.01	0.0932	0.3053	66%
Adding Chu et al. Respirology. 2016 (k=18)	1.53	[1.27; 1.85]	< 0.01	0.0923	0.3038	65%
Adding Elias et al. Sleep Med. 2016 (k=19)	1.50	[1.24; 1.81]	< 0.01	0.0940	0.3066	64%
Adding Xu et al. Sleep Med. 2016 (k=20)	1.49	[1.25; 1.79]	< 0.01	0.0878	0.2963	62%
Adding Yeo et al. Ann Transl Med. 2017 (k=21)	1.47	[1.25; 1.73]	< 0.01	0.0760	0.2756	61%
Adding Mehta et al. Bone. 2018 (k=22)	1.44	[1.23; 1.68]	< 0.01	0.0750	0.2739	61%
Adding Yatsu et al. Clin Res Cardiol. 2018 (k=23)	1.47	[1.26; 1.72]	< 0.01	0.0804	0.2835	63%
Adding Koo et al. Heart. 2020 (k=24)	1.48	[1.28; 1.72]	< 0.01	0.0753	0.2745	62%
Adding Ding et al. Int. J. Environ. Res. Public Health. 2021. (k=25)	1.49	[1.28; 1.72]	< 0.01	0.0728	0.2699	60%
Adding Wang et al. BMJ Open. 2021 (k=26)	1.48	[1.28; 1.71]	< 0.01	0.0704	0.2654	59%
**Random effects model**	**1.48**	**[1.28; 1.71]**	**< 0.01**	**0.0704**	**0.2654**	**59%**

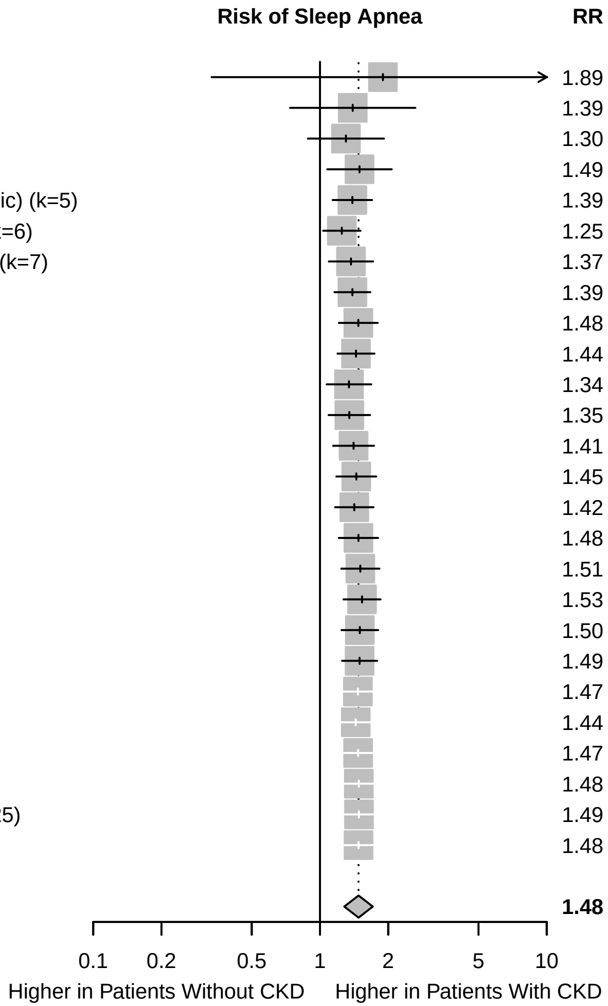

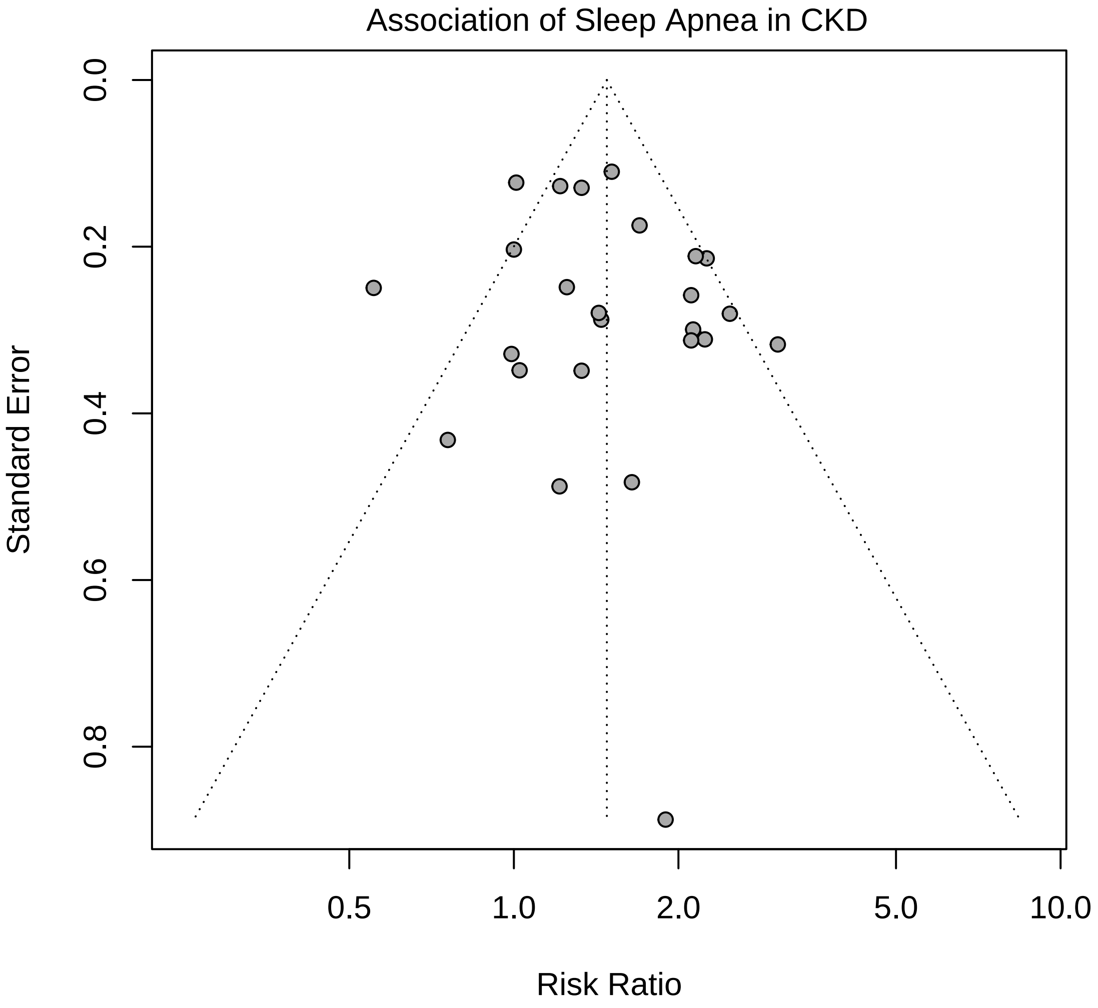

Association of Sleep Apnea in CKD
Standard Error
0.0
0.2
0.4
0.6
0.8
Risk Ratio
0.5
1.0
2.0
5.0
10.0
