# Supplementary material for: Bidirectional association of sleep disorders with chronic kidney disease: a systematic review and meta-analysis
Source: Clin Kidney J. 2024 Oct 18;17(11):sfae279. doi: 10.1093/ckj/sfae279 (PMC11549560; doi:10.1093/ckj/sfae279)
Supplement: sfae279_Supplemental_Files [file sfae279_supplemental_files.zip › S6. GRADE.docx]

Supplement 6. Evaluation of quality of pooled evidence using the Grading of Recommendations Assessment, Development and Evaluation (GRADE) framework.

| Outcomes | Effect size (95% CI) | Number of patients (number of included studies) | *I^2^* | A | B | C | D | E | F | G | H | Quality of evidence |
| --- | --- | --- | --- | --- | --- | --- | --- | --- | --- | --- | --- | --- |
| Risk of CKD in the presence of OSA | 1.68 (1.45 to 1.93) | 23, 983, 194 (26) | 44 |  |  |  |  |  |  |  |  | Moderate |
| Risk of CKD in the presence of sleep apnea | 1.66 (1.39 to 1.98) | 699, 092 (18) | 64 |  |  |  |  |  |  |  |  | Moderate |
| Risk of CKD in the presence of RLS | 1.88 (1.48 to 2.38) | 25, 972 (7) | 52 |  | -1 |  |  |  |  |  |  | Low |
| Risk of CKD in the presence of insomnia | 1.24 (1.01 to 1.54) | 2, 153, 816 (7) | 0 |  |  |  |  |  |  |  |  | Moderate |
| Risk of CKD in the presence of other sleep disorder | 1.80 (1.41 to 2.32) | 132, 460 (5) | 34 |  |  |  |  |  |  |  |  | Moderate |
| Risk of OSA in the presence of CKD | 1.77 (1.56 to 2.01) | 23, 983, 012 (29) | 37 |  |  |  |  |  |  |  |  | Moderate |
| Risk of sleep apnea in the presence of CKD | 1.56 (1.32 to 1.84) | 708, 958 (20) | 59 |  |  |  |  |  |  |  |  | Moderate |
| Risk of RLS in the presence of CKD | 1.73 (1.32 to 2.25) | 24, 856 (8) | 56 |  | -1 |  |  |  |  |  |  | Low |
| Risk of insomnia in the presence of CKD | 1.14 (1.03 to 1.27) | 2, 153, 816 (7) | 27 |  |  | -1 |  |  |  |  |  | Low |
| Risk of other sleep disorder in the presence of CKD | 1.45 (1.01 to 2.08) | 132, 522 (8) | 69 | -1 |  |  |  |  |  |  |  | Low |

A: risk of bias among included studies. B: imprecision. C: inconsistency. D: indirectness of evidence. E: publication bias. F: dose response gradient. G: large effect size. H: biases increasing confidence in the estimate.
