# Supplementary figures and images for: Bidirectional association of sleep disorders with chronic kidney disease: a systematic review and meta-analysis
Source: Clin Kidney J. 2024 Oct 18;17(11):sfae279. doi: 10.1093/ckj/sfae279 (PMC11549560; doi:10.1093/ckj/sfae279)

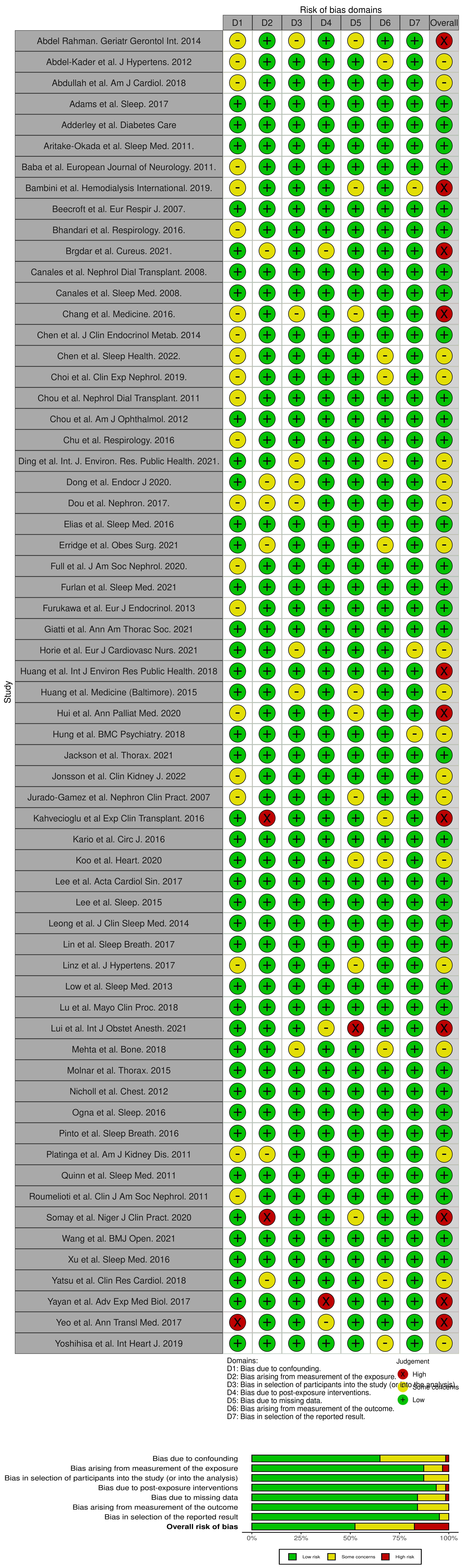

Supplement: sfae279_Supplemental_Files [file sfae279_supplemental_files.zip › S2. ROBINS-E.tif]
